# Establishing a Provincial Registry for Recurrent Prostate Cancer: Providing Access to PSMA PET/CT in Ontario, Canada

**DOI:** 10.3389/fonc.2021.722430

**Published:** 2021-08-02

**Authors:** Sympascho Young, Ur Metser, Golmehr Sistani, Deanna L. Langer, Glenn Bauman

**Affiliations:** ^1^London Regional Cancer Program, Department of Oncology, Western University and London Health Sciences Centre, London, ON, Canada; ^2^Joint Department of Medical Imaging, Princess Margaret Hospital, University Health Network, Mount Sinai Hospital, Women’s College Hospital and University of Toronto, Toronto, ON, Canada; ^3^Department of Medical Imaging, London Health Sciences Center and Western University, London, ON, Canada; ^4^Clinical Institutes and Quality Programs, Ontario Health (Cancer Care Ontario), Toronto, ON, Canada

**Keywords:** PSMA - prostate specific membrane antigen, registry, prostate cancer, biochemical failure (BF), positron emission tomography (PET), Ontario (Canada), health policy, healthcare funding

## Abstract

Prostate Specific Membrane Antigen (PSMA) positron emission tomography/computed tomography (PET/CT) is becoming established as a standard of care for the (re)staging of high-risk primary and prostate cancer recurrence after primary therapy. Despite the favorable performance of this imaging modality with high accuracy in disease detection, the availability of PSMA PET/CT varies across jurisdictions worldwide due to variability in the selection of PSMA PET/CT agent, regulatory approvals and funding. In Canada, PSMA based radiopharmaceuticals are still considered investigational new drug (IND), creating limitations in the deployment of these promising imaging agents. While regulatory approval rests with Health Canada, as a single payer health system, funding for Health Canada approved drugs and devices is decided by Provincial Health Ministries. Ontario Health (Cancer Care Ontario) (OH-CCO) is the agency of the Ministry of Health (MOH) in Ontario responsible for making recommendations to the MOH around the organization and funding of cancer services within Ontario (population of 15 million), and the PET Steering Committee of OH-CCO is responsible for providing recommendations on the introduction of new PET radiopharmaceuticals and indications. For Health Canada approved PET radiopharmaceuticals like 18F-FDG, OH-CCO (on behalf of the MOH) provides coverage based on levels of evidence and specific PET Registries are established to aid in real-world evidence collection to inform OH-CCO regarding emerging PET applications. In the case of PSMA PET/CT, adapting this model to an IND PSMA PET/CT agent, 18F-DCFPyL, necessitated the creation of a hybrid Registry-Study model to leverage the existing OH-CCO Registry structure while respecting the need for a Health Canada Clinical Trials Application (CTA) for the deployment of this agent in the province. Within the first 2 years of the registry, over 1700 men have been imaged resulting in a change in management (compared to pre-PET management plans) in over half of the men imaged. In this article, we describe the organization and deployment of the PSMA PET/CT (PREP) Registry throughout the province to provide access for men with suspected prostate cancer recurrence along with key stakeholder perspectives and preliminary results.

## Introduction

Ontario has a publicly funded health care system with a proven track record in clinical trials, health services research and evidence-based medicine (1). Two decades ago, when FDG PET was rapidly adopted as a new clinical tool in various jurisdictions worldwide, Ontario adopted a more cautious approach (2). To address limitations in the literature that PET scanning impacted patient management decisions and outcomes, several high-quality randomized clinical trials were launched. As evidence matured, funding for PET as an insured service was provided for the specific clinical indications where PET was clinically beneficial and had advantages over other testing. An initial government advisory board became the Ontario Steering Committee for PET Evaluation (“PET Steering Committee”), which was initially assigned the task of reviewing of the literature and generating some of the needed evidence by undertaking a series of trials. For indications where the existing evidence for the use of PET was limited but compelling (e.g., retrospective studies suggestive of impact to care), PET Cancer Registries were established (2). The registries facilitated real-world evaluation and evidence-building in the Ontario context for specific clinical indications, enabling access to PET scanning for patients while collecting a minimum dataset (such as pre- and post-PET stage, pre- and post-scan intended treatment) that could then be linked to provincial administrative databases to determine a change in management decisions, actual treatment delivered, and patient outcomes after the provision of PET (3). While praised as an evidence-based approach to ensure funded interventions demonstrate clear benefits to patients, this model has also been criticized by others as perhaps too rigorous (unreasonable to expect diagnostic tests to show impact on clinical outcomes where downstream management strategies might diverge quickly confounding the influence of imaging on outcomes) and that the time it takes to acquire high-level evidence may limit patient access to new PET technologies (2).

The present provincial PET Steering Committee, currently an Ontario Health (Cancer Care Ontario) (OH-CCO) committee, has a mandate to provide recommendations on the clinical indications for use, quality criteria, and distribution and access to PET scan services. The Committee continues to assess potential PET indications through multiple mechanisms, including: 1) proactive systematic literature reviews supported by the Program in Evidence-Based Care (cancercareontario.ca/en/guidelines-advice), which involve reviews of all clinical practice guidelines as well as primary literature of high-quality PET trials; 2) ongoing evidence-building through PET Registries; 3) provincial-level support for clinical trials, including a limited number of randomized controlled trials (NCT02751710, NCT02462239) if PET Registry-type data would not suffice to address questions of utility or outcome. When identified, new potential PET indications are discussed together with disease-site experts from the relevant OH-CCO Ontario Cancer Advisory Committees to determine whether the available evidence is sufficient to make a recommendation for funding of the new indication as an insured service or whether further data is needed through a clinical trial or PET cancer registry.

In 2016, ^18^F-DCFPyL, an ^18^F-labeled second generation PSMA tracer, became available for use in Ontario through clinical trials (4). Multiple investigator-led trials evaluating the use of PSMA PET in prostate cancer were launched, predominantly for restaging men at the time of biochemical failure (NCT02856100, NCT02793284). Awareness of the availability of PSMA PET in these trials and the increasing reports of lesion-directed therapy, radiotherapy, and surgery, for patients with oligometastatic disease, also led to increased demand for access to PSMA PET outside of these trials. This, along with emerging reports in the literature on the diagnostic accuracy and clinical impact of PSMA PET (5–8), incentivized the development of a prospective Provincial PSMA PET Registry Study in collaboration with the provincial Genitourinary Advisory Committee at OH-CCO. This Registry Study would utilize existing provincial infrastructure to support access to PSMA PET for recurrent prostate cancer, compliant within the Health Canada regulatory framework, in several scenarios of suspected persistent or limited recurrent disease after primary therapy at various decision points in the disease trajectory. In addition to patient access, the design supports consistent, large scale real-world data collection to inform where PSMA PET is the most impactful in detecting sites of disease recurrence and guiding management; this data, in turn, can be leveraged to refine which indications are recommended for routine funding.

## Methods

### Establishing the Registry Study

In order to establish the Registry Study, a common provincial clinical trial protocol was developed (**Supplementary Material**s) with co-primary investigators from Nuclear Medicine, Radiation Oncology and Uro-Oncology. The protocol provided for investigation of PSMA PET/CT across a variety of clinical scenarios in the setting of prostate cancer recurrence after primary therapy, after PET/CT directed therapy, or through an access cohort for PET/CT-assisted decision making in scenarios not covered by the other cohorts. Minimum sample sizes for each cohort were determined based on performance for lesion detection by PSMA PET/CT as reported in the literature. Among the provincial cancer centers that had expressed the interest and capacity to participate in the Registry Study, a lead center and overall project coordinator at that center were identified to initiate the regulatory approval process for the Province. From the clinical protocol, the lead center obtained a Health Canada CTA for the use of the PET/CT tracer and the protocol was submitted by the lead center to the Ontario Cancer Research Ethics Board (OCREB), a centralized Ethics Review board that is recognized as “Board of Record” by all the participating sites. Following OCREB approval for the lead center, other individual sites applied through OCREB for approval as a participating center with a site Principle Investigator.

Each participating center assigned a multidisciplinary group of collaborators and a local lead investigator, recruited study coordinators for screening, consenting and scheduling patients and for maintaining all regulatory and other study documents in collaboration with the study sponsor. The new registry utilized experience from prior registries as well as the expertise built through investigator-led PSMA PET trials, existing PET/CT infrastructure in the province, and centralized radiopharmaceutical production. ^18^F-labeled PSMA radiopharmaceuticals were chosen instead of ^68^Ga due to its advantages in the setting of a large multicenter registry (in a province more than 1.5 times the size of the state of Texas). First, ^18^F-labeled radiopharmaceuticals are produced at a cyclotron facility, rather than with a ^68^Ge/^68^Ga generator. This enables central radiopharmaceutical production and participation of multiple PET centers without needing to procure multiple generators and/or rely on local radiopharmaceutical production. Second, the larger volume of radiopharmaceutical produced in a cyclotron along with the longer half-life of ^18^F compared to ^68^Ga (110 minutes vs 68 minutes, respectively) facilitates distribution to distant centers across the province. Patients are booked for PSMA PET after securing a dose on a provincial roster for upcoming radiopharmaceutical production days. The number of production days is adjusted according to demand. For those centers located within Southern Ontario, distribution of radiopharmaceutical by land transportation was feasible with central production occurring in the morning, followed by transportation of the radiopharmaceutical and imaging at the regional PET centers occurring in early to late afternoon. One site (Ottawa) was primarily served *via* air transport, but had land transportation as a back-up option if required. One site in Northern Ontario (Thunder Bay site) was supplied exclusively by air transportation.

In order to gauge the effectiveness of PSMA PET/CT compared to conventional imaging, the initial phase of the Registry Study required all men to be staged with conventional imaging (bone scan and CT) prior to PSMA PET/CT. Men were eligible for PSMA PET/CT if the conventional imaging demonstrated either no lesions, equivocal lesions or less than four metastases (oligometastatic disease). Reads were conducted by local readers with no centralized read, however informal support for challenging cases was provided through peer-to-peer consultation. Post-PSMA PET/CT results were provided back to the referring physicians and completion of a change in management questionnaire based on the PSMA PET/CT results was required. Information sent back centrally to OH-CCO included standardized reporting of the PSMA PET/CT and post management questionnaires. Existing provincial payment mechanisms for PET/CT were utilized to reimburse participating centers for the costs of conducting the PSMA PET/CT (tracer and technical costs and physician reads). Recognizing the additional workload associated with the Registry Study required in order to be compliant with Health Canada regulatory requirements, participating centers received support for related activities (e.g. patient eligibility and consent, documentation, data submission). Phase II of the Registry Study was launched in September 2020 and removed the requirement for pre-PET/CT conventional imaging for men with PSA <10 ng/ml at the time of imaging given the low yield of conventional imaging at lower PSA levels (9).

### Key Stakeholder Interviews

Given the unique “hybrid” nature of the registry study, we conducted targeted structured interviews with key stakeholders (investigators, administrative personnel, study personnel, patients) to identify benefits and strengths of this hybrid approach as well as identify gaps and weaknesses after completion of the first phase of the Registry Study. Interviews were conducted through videoconferencing using a semi-structured interview guide. Interviews were recorded and reviewed for coding and qualitative analysis.

## Results

### Accrual and Preliminary Study Results

**Figure 1:**
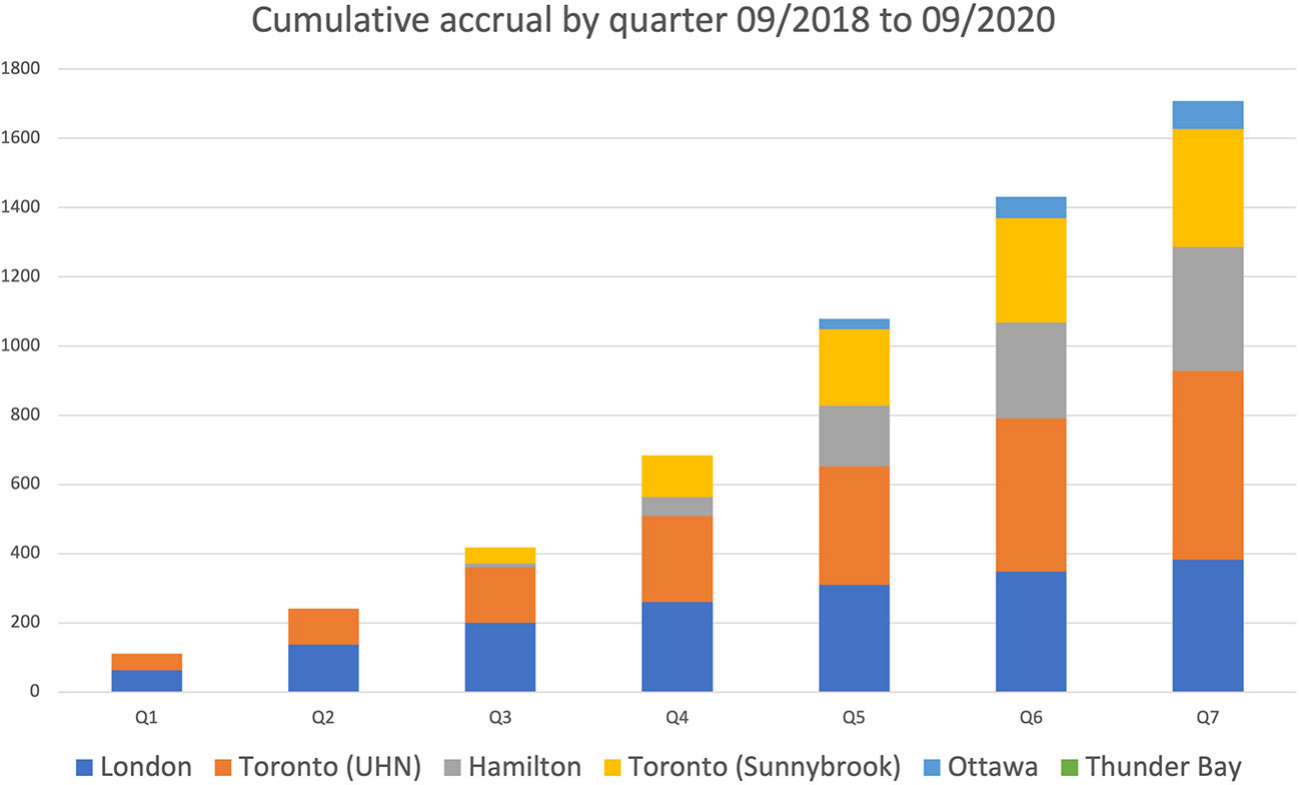
Location of participating PREP Centers.

**Figure 2:**
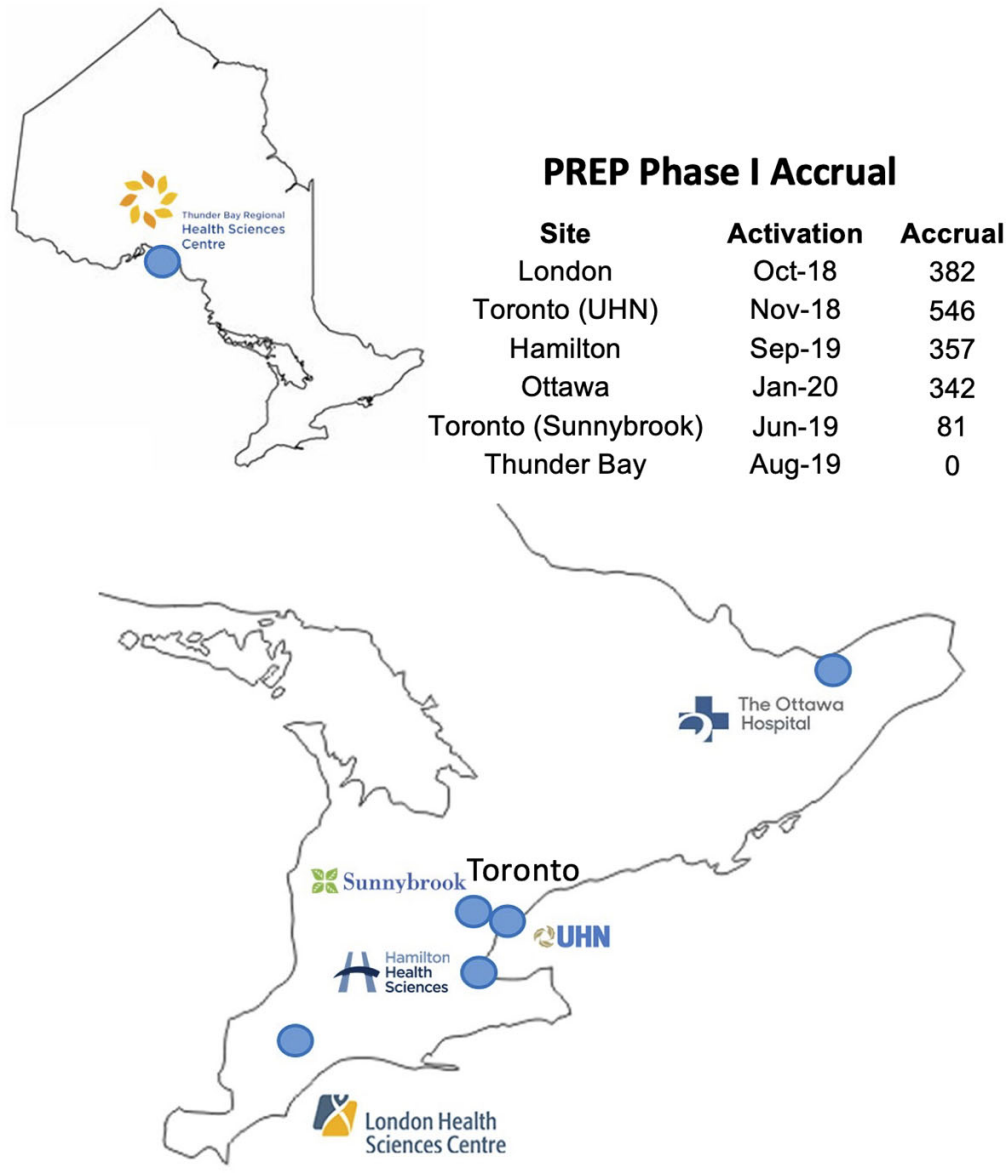
Accrual by quarter across PREP Centers.

After receiving regulatory approvals from OCREB and Health Canada, the PSMA-PET for Recurrent Prostate Cancer (PREP) registry was launched in September 2018 and included 5 participating PET centers across the province (**Figure 1**). Men were eligible for enrollment based on predefined clinical scenarios/cohorts (**Table 1**). Recruitment to the registry study was swift with over 1700 patients scanned in the first 21 months (Phase I of the registry) (**Figure 2**). The majority of referrals were from urology and radiation oncology, with a minority of referrals from medical oncology. In October 2020, Phase II of the Registry was launched; the major refinement being removing the requirement for restaging CT and Bone Scan for men with PSA < 10 at the time of enrollment. There was one adverse event reported during Phase I of the Registry, this was deemed unrelated to the radiotracer itself. A small percentage of cases (<5%) planned scans needed to be rescheduled because of a failure of production run of tracer from the centralized distribution site. Among successful production runs, there was one instance of a missed sterility test which resulted in the need to reschedule the planned scans as the product was not administered.

**Table 1:** PREP Registry Cohorts.

Cohort	Description
1	Men with node positive disease or detectable PSA within 3 months of prostatectomy
2	Men with biochemical failure (PSA >0.1ng/ml) post prostatectomy
3	Men with biochemical failure (PSA >0.1ng/ml) post prostatectomy following adjuvant or salvage pelvic radiotherapy
4	Men with biochemical failure post prostatectomy and salvage hormone therapy (with or without salvage/adjuvant radiotherapy)
5	Evaluation of response among men with PSMA PET/CT directed treatment
6	Men with biochemical failure (PSA >2.0ng/ml/Phoenix criteria) post radiotherapy
7	Access cohort for PSMA PET/CT assisted decision making in men not meeting criteria for Cohorts 1-6

Overall, nearly two-thirds of PSMA PET scans were positive, including > 60% of studies performed in patients with negative CT and bone scintigraphy. As reported in prior studies, the detection rate of PSMA PET increased with level of serum PSA at time of inclusion. Nearly a third of patients had evidence of locoregional failure on PET. A quarter of patients had oligometastatic disease, defined as up to 4 sites of disease, and nearly 10% had extensive metastases detected on PET. The high detection rate of additional disease by PSMA PET in men with suspected low volume metastatic disease resulted in frequent changes in management.

### Stakeholder Feedback

Seventeen key stakeholders (5 referring physicians from urology and radiation oncology, 4 nuclear medicine physicians, 6 research coordinators and 2 patients) were interviewed. Participant responses were grouped into themes, which are described below.

#### Successes of the Study

Physicians, study coordinators and patients were overwhelmingly positive about the value of PSMA PET/CT scans. Physicians expressed that the access to PSMA PET/CT scans has been “game changing” and given valuable information, clarity and more assurance in managing recurrent prostate cancer. One urologist (referring physician #4) revealed that it has changed prostate cancer management at their center to such a degree that “We now basically have PSMA PET rounds instead of tumor boards. We’re discussing the significant of PSMA studies for every patient case.” When inquired about PSMA’s impact on prostate cancer management, he described that “It feels like we have to start over and figure out how to manage prostate cancer again. I thought I knew how to manage prostate cancer until PSMA PET came along.” From the perspective of nuclear physicians, several noted that the Registry Study also provided an excellent opportunity to develop and enhance their skills in interpreting PSMA PET/CT. Nuclear physicians were able to easily integrate PSMA PET into existing PET/CT workflows. Patients accessing the registry were similarly positive, felt well-supported by staff and describe the intake process as efficient. For one patient, “the test took only two hours, and was easier or comparable to CT, nuclear, and MRI tests experiences that I’ve received recently. Further, having more confidence in the fidelity of the results, it is allowing me to explore my continuing treatment strategies and paths forward with better information on the state of my disease.” (Patient #2) Another patient commented that PSMA PET “highlighted some cancerous cells were lurking [in my lungs] and allowed my doctor the ability to plan a course of treatment very quickly.” (Patient #3).

Overall, referring physicians and study coordinators acknowledge that enrolling patients in the Registry study involved some paperwork, though attitudes towards the paperwork were mixed. Most referring physicians felt that it was relatively straight-forward to enroll patients, “not onerous” and that they received plenty of support from the lead study site when they encountered issues. One urologist commented “it’s nothing, it takes a couple of seconds.” On the other hand, there were physicians who resented the additional paperwork involved and felt the Registry Study was “cumbersome for the value of the science” and a limiting factor for patient access. After enrolling patients, physicians agreed unanimously that imaging results were easily viewable in the existing electronic medical records (EMR) at their centers.

#### Drawbacks of a Study Approach

Though stakeholders were generally positive about the study, with most physicians recognizing that the registry study served in part to improve provincial access to PSMA PET, there was still the feeling that Ontario lagged behind certain parts of the world in respect to PSMA PET. One of the criticisms was a sense that there is already enough international evidence supporting the use of PSMA PET in these patient populations, and thus limited benefit in gathering additional registry-style data at the cost of introducing barriers for both physicians and patients. Other respondents, however, welcomed the registry study as generating needed information: “The most important thing that will come from this study is defining the population of men that PSMA PET/CT scans are most beneficial for, and the PSA thresholds when the scan is beneficial.” One urologist felt the study approach was a way for Ontario to “limit [expenses and] access to PSMA PET by creating hurdles” for clinicians to order the test, perhaps not realizing that without the Registry study framework in place to address Health Canada regulatory requirements, the provision of PSMA PET as a clinical service would not be possible.

One of the barriers of the study approach was that due to the ethical policies around clinical study consenting, physicians at most centers could not obtain consent from their own patients. As a result, study coordinators were required to obtain consent from patients. Paperwork requiring details of prior treatments, radiation, and post-imaging forms were sometimes described as “cumbersome” and “tedious” by busy clinicians. On the other hand, study coordinators mentioned that often spent considerable time tracking down physicians for post management forms and to “make sure all of our referral forms are filled in correctly.” The requirement for a bone and CT scan within 3 months of the PSMA PET were also seen unanimously as a barrier by referring physicians and study coordinators, which led to additional costs of “unnecessary scans” and delays in the ability to enroll patients on trial, as wait-times for conventional imaging could add weeks or months to accessing PSMA PET/CT. Phase II of PREP was able to mitigate this concern, as the bone scan and CT criteria was removed.

#### Access to Regional Cancer Centers

Another drawback of the study approach is that only physicians who were co-investigators of the study could enroll patients onto the registry. At participating centers, most physicians in either radiation oncology or urology were involved as co-investigators of the study and could directly enroll patients. However, physicians in non-participating centers could not directly enroll their patients and were required to refer patients to co-investigators of the study in order for their patients to be enrolled. As a result, one or two physicians at each center are referred the majority of these patients and facilitate scans for them. This supports broader patient access, but was identified to be inefficient for patients, their referring physicians, and the designated co-investigators at each center.

#### Production and Distribution of Radiopharmaceuticals

Central production of radiopharmaceuticals was identified to have both advantages and disadvantages. While central production offered the advantages of greater production efficiency, cost-effectiveness and easier licensing, it also made the supply chain more vulnerable to transportation and production issues. For instance, since the entire province was supplied by a single cyclotron, when radiopharmaceutical production issues inevitably occurred, all participating centers were affected and scans across the province had to be cancelled. These uncommon but last-minute cancellations of scans led to additional patient frustration and anxiety.

In addition, distribution from a central source made the supply chain vulnerable to transportation challenges. For the Thunder Bay site, distribution through commercial air travel created additional several months-long delays in setting up the study, as additional waivers had to be obtained from the Ministry of Transportation to enable delivery. Unfortunately, this delay led to “a lot of upset and anxious patients because they had signed the consent forms and were waiting and waiting to get the scan to direct their cancer treatment … and for several months, [physicians] could not offer patients the scan and we had to refer patients to Toronto.” (Radiation oncologist, referring physician #5).

With any new supply chain, estimating and developing an appropriate production capacity is challenging. When the study was first activated, there was high patient enrollment but inadequate radiopharmaceutical production and distribution capacity. A study coordinator commented that even in the first month of the study, “we were already starting to get a backlog of patients” due to lack of radiopharmaceutical supply. Over time, additional production days were added at the cyclotron to accommodate clinical need. Study coordinators are now satisfied with the supply: “wait times are caught up” and “the access radiopharmaceuticals is no longer limiting the amount of scans per month.”

#### Patients Wait Times

Wait times for scans were dependent on both radiopharmaceutical availability as well as PET scanner availability. As previously noted, availability of ^18^F-DCPyL was more of a limiting factor early on in PREP, and impacted by both the number of production days supported and the occurrence of production issues leading to cancelled bookings. As a Registry Study, PSMA PET scans were also provided during regular clinical hours (i.e., there were not additional dedicated hours), and these scans needed to be incorporated as part of clinical demand. As such, wait times for PSMA PET were also subject to the regular operating pressures experienced by the participating PET centers, and prioritized accordingly. Notably, at two of the centers (London and Hamilton), PET scanners were down for a number of weeks, creating a backlog of patient scans. Wait times for PSMA PET scans have been 6-8 weeks on average for patients in the London, Hamilton, Toronto and Ottawa centers. For reference, these are longer than wait times for FDG PET scans in Ontario, given the difference in clinical urgency compared to most routine PET scans as well as dictated in part by having limited PSMA scan days each week.

#### Impact of the COVID-19 Pandemic

At most centers, the enrollment of patients onto the Registry Study was not significantly impacted by the restrictions to clinical trial activities during the pandemic as the Registry was regarded as “clinically essential” research. However, the pandemic’s impact was felt disproportionately at the Thunder Bay site, as it affected the availability and predictability of commercial air travel, leading to frequent cancellations and inability to reliably transport radiopharmaceuticals by air. Radiopharmaceutical availability was described as a “gong show” and both anxiety-provoking for patients who had to be constantly rescheduled and frustrating for research coordinators and physicians. Unfortunately, Thunder Bay was forced to cancel scans altogether on January 29^th^, 2021 and stop further accrual of patients due to the state of air travel. Patients already enrolled had to either travel to Toronto or out of the country for scans, or simply decided to go ahead with treatment based on results of conventional imaging. A physician at Thunder Bay observed that “central production and distribution are not a sustainable setup if the trial will go on for several years. It puts us at the mercy of available transportation, which is an ever-changing situation with COVID.” (Radiation oncologist, referring physician #5). Though unfortunate, this experience is similar to others in Europe and Asia, where the pandemic also heavily impacted nuclear medicine departments and delayed radiopharmaceutical supply (10).

#### Finance and Funding Process

The hybrid Registry-Study model proved challenging in terms of the flow of funding. Consistent with the Ontario Ministry of Health (MOH) directive that guides transfer of funding for organizations such as OH-CCO, funding was provided to participating sites for specific deliverables (e.g., fulfilling Health Canada regulatory and ethics board requirements, volume funding for the PSMA PET scans, data submission), rather than for identified roles or units of time, etc. And, as these PSMA PET scans were provided as part of the overall provincial PET program, funding was managed *via* the existing agreements supporting clinical PET scanning services. Agreements are also issued for each fiscal year, with execution occurring within-year; funding is then initiated at the time of execution, to be retroactive to the beginning of fiscal. Several stakeholders, including physicians and study coordinators, commented that this approach – which differs from that of traditional clinical trials – led to operational challenges. All participating sites had dedicated research units with well-defined processes, including approaches for budgeting and contract format expectations (e.g., total-budget over multiple years, versus annual agreements) for costs associated with the conduct of individual cancer clinical trials. Funding was also managed at the level of the institution versus research unit, and did not include delineated budgets for research study coordinators or other resources. Internal institutional processes were also needed to manage transfers between departments which, from the perspective of the research units, made it “difficult to follow where the money [for research] went.” This led to difficulties in getting approvals to hire and pay for coordinators, and, at one site an experienced investigator who was interested in leading the study at their site was unable to do so due to because of issues related to funding and processes. In one of the years of the Registry study there was a significant delay in issuing the agreement and subsequent delay in the release of funds and transfer to the research unit. At one site this caused significant challenges in managing staffing from within the designated budget at the institution; a nuclear physician commented that “The funding for our research coordinator was still missing after a year; if not for alternate sources of funding, our research coordinator would not have been paid.” The unique needs of a Registry study utilizing an IND agent added consenting and regulatory requirements that fell outside the usual functions of the PET centers and added to the complexity of budgeting and funding of PREP activities.

#### Limitations of ^18^F-DCFPyL Radiopharmaceutical

Generally, performance of ^18^F-DCFPyL within PREP was on par with results reported for ^18^F-DCFPyL by other institutions and other PSMA PET agents like ^68^Ga-PSMA. Both radiation oncologists and nuclear physicians observed that the pharmacokinetics of the specific PSMA agent, ^18^F-DCFPyL, had limitations for use in the PREP indications as the primary route of GU excretion could interfere with detecting local recurrences (prostate bed and prostate). Adopting PSMA agents with hepatic excretion was felt to be potentially helpful for the future, particularly for those patients with earlier recurrence.

#### Nuclear Medicine Physician Training for Interpreting PSMA PET/CT Scans

For nuclear medicine (NM) physicians, the Registry also provided an opportunity to enhance their skills in interpreting PSMA PET/CT. In general, each center had at least one NM physician who had prior exposure to PSMA PET/CT interpretation, either through past practice at a center with PSMA PET/CT, through participation in prior PSMA PET/CT clinical trials, or in the review of PSMA PET/CT for patients from their center referred out of province for imaging. These individuals helped organize local initiatives such as peer to peer mentoring through grand rounds, case discussion forums or encouraging the adoption of online training to ensure other members gained proficiency in scan interpretation. PSMA lectures at conferences (regional and national) helped all teams to become more familiar with PSMA PET imaging and investigators taking part in the Registry were involved in helping organize these conferences.

#### Unexpected Impacts

An unexpected clinical impact of PSMA PET scans was noted among men with biochemical failure post-radical prostatectomy and negative or equivocal scans. These patients were often reluctant to undergo salvage treatment (standard of care), and have opted instead to be followed with surveillance. One urologist explained “you cannot get patients to do salvage radiotherapy, because they’ll say “What are you going to radiate? There’s nothing on the PET scan!” And then you’ll have to teach them about sensitivity/specificity which may be difficult to accomplish in a clinic visit. It is easier to see these patients in close follow-up rather than send them for salvage RT.” The urologist also predicted that with more patients undergoing PSMA PET scans at low PSAs, “I’ll bet salvage radiotherapy rates are going to go down significantly.” Given that failure free survival from salvage RT are highest among those patients with absent or prostate fossa restricted PSMA PET/CT uptake (11), this strategy may not be the most appropriate. Further research and education of both clinicians and patients regarding this clinical scenario are important.

An unexpected system level impact of the study is that it created a useful pipeline and network between treating physicians, nuclear medicine physicians and PET centers that did not previously exist in Ontario. This has created an infrastructure for the development of future projects, such as the CPD-002 (NCT04644822) and PATRON trials (NCT04557501).

## Discussion

This paper has outlined the process and initial outcomes of launching a Registry study within the province of Ontario, Canada. The PREP registry was launched as a pragmatic response in order to (1): Enable access to advanced prostate cancer imaging with PMSA PET/CT on a provincial scale (multi-center across Ontario) (2) build evidence to inform the most appropriate and impactful indications for PSMA PET and (3) support the nuclear medicine community in gaining experience with this radiopharmaceutical (12). Based our stakeholder feedback conducted, though there were challenges, the Registry successfully addressed all three aims.

With regards to access, PSMA PET is currently not standard of care in Canada, and aside from access through the PREP registry, there are no prostate cancer-specific PET radiopharmaceuticals approved for routine clinical use by Health Canada. This is similar to much of the world currently, in that access to PSMA PET is still limited and only available through clinical studies. As the evidence continues to build, policies are quickly changing. For example, in the United States, the first PSMA PET radiotracer 18F-DCPyL was approved by the FDA for commercial use on May 27, 2021 based on findings from prospective phase 2/3 trials OSPREY and CONDOR (13, 14).

Australia, one of the world leaders in PET, took a different approach to regulating radiopharmaceuticals. When PET/CT imaging was initially registered with the Australian Therapeutic Goods Administration (TGA), it came with the approval to use any PET radiopharmaceuticals (15). As a result, new radiopharmaceuticals do not go through the same regulatory mechanisms as other pharmaceuticals (15). This regulatory landscape allowed early roll out and adoption of PSMA PET technology by Australian physicians, as early as 2014. By 2015, PSMA PET became the primary mode of primary and secondary staging of prostate cancer (>90% of all patients) at an Australian center, despite a lack of clinical evidence supporting its use at the time (16). This approach has both pros and cons, and balancing the tradeoffs between the benefits of early adoption and threshold of evidence required is something that every public-health system must decide for itself. However, in this case Australia’s regulatory policies have clearly allowed the advantage of early adoption and widespread access of a valuable diagnostic modality. In a cost-effectiveness analysis of the proPSMA study, PSMA PET was modeled to be more cost effective than CT and bone scans in the Australian setting (17). Similarly, in Germany, a permissive regulatory environment has fostered an environment favoring innovation in PSMA based theranostics, however, there is variable access to these agents both by indication and by jurisdiction (18). Whether the same economics hold true in Canada remains to be seen and the PATRON (NCT04557501) trial plans to conduct a cost-effectiveness analysis in the Canadian setting as well as tracking clinical impact of PET informed treatment.

Beyond regulatory approval, in order for patients to access PSMA PET in a single-payer public healthcare system such as Canada’s, there needs to be a funding mechanism to support clinical use in the appropriate indications. In Canada, such funding is organized at the provincial level, and for PET in Ontario, OH-CCO reviews and recommends funding through an evidence-based process in order to maximize health care investment in areas where there is strong evidence supporting clinical impact and patient and/or system benefit; the Ministry of Health, in turn, must prioritize investments across the health-care system. In considering radiopharmaceuticals such as PSMA PET-based agents, generating evidence to satisfy both regulatory and funding decisions can be challenging. Regulatory decisions for a new diagnostic agent/test are based primarily on considerations of safety and test accuracy. In the case of prostate cancer, patterns of disease recurrence tend to be in locations that are less accessible to biopsy (i.e., pelvic or para-aortic lymph nodes, bone) and, as a consequence, reliance on clinical surrogates such as correlative imaging or response to therapeutic interventions may be necessary (19). In order for PSMA PET to be approved for funding, more stringent levels of evidence may be necessary, such as clinical trials that demonstrate an impact on patient outcomes, consider cost efficacy and/or benefits over other testing. Such endpoints are challenging to demonstrate in prostate cancer, where a long natural history and multiple therapeutic interventions can obscure the long-term impact of early diagnostic decision points on endpoints like metastases free or overall survival. Nevertheless, randomized trials examining clinical endpoints like biochemical disease-free survival after PET directed salvage therapy post prostatectomy are underway (Quebec phase II trial NCT03525288 and pan-Canadian PATRON trial NCT04557501, Swedish trial NCT04794777, Netherland’s trial PERYTON NCT04794777, and UCLA’s PSMA SRT NCT03582774) and may provide the evidence base to inform funding decisions.

In an effort to gather real world evidence using a cost-effective strategy, the PREP Registry Study utilized existing PET Registry processes managed by OH-CCO as part of the provincial PET program. Many of the identified challenges and barriers stemmed from the hybrid Registry Study model of PREP, which was necessitated by the absence of Health Canada approval for PSMA agents and requirement of a Health Canada CTA. Previous (and ongoing) OH-CCO Registries building evidence for emerging clinical evidence for indications of FDG PET did not encounter the same challenges, primarily because FDG is approved by Health Canada. However, the clinical data collection for FDG PET Registries that is required to strengthen and build evidence in the Ontario setting of care - aligned with the goal of data collection in PREP - can also be perceived as a burden for busy clinicians and PET administrative teams. Although overall positively received as an approach to bring PSMA PET scans to patients, the hybrid model also created inherent challenges identified through our stakeholder interviews in activating and conducting the Registry. While funding was provided to support sites in meeting the trial requirements for the PSMA PET scans to occur, clinical trial functions such as trial activation and regulatory approvals like REB submissions and patient enrollment and consenting were often managed outside of clinical operations, through separate clinical trial research units (CRUs). Achieving the goals of the Registry study, including meeting regulatory and clinical requirements aligned with funding deliverables, thus required significant collaboration between research and clinical departments. The funding approach employed by OH-CCO provides for flexibility in how sites accomplish the goals, but internal agreement on roles and reimbursement for the CRUs for their contributions to the PREP registry tasks needed to be organized on a per-center level. In many cases this created delays in trial activation in some centers due to negotiations between the PET centers and the CRUs. Additionally, Health Canada regulations required referral of patients to centers participating in the PET Registry Study, even for regions with local access to PET/CT. This requirement created additional hurdles for access for men outside of the PREP Centers and additional workload for the PREP Centers themselves as men referred for PSMA PET/CT would need to be consented by physicians at the PREP Centre for the Registry study. While telemedicine was utilized in many centers to address this hurdle, this inefficiency will persist as long as Health Canada approved PSMA PET radiopharmaceuticals are not available.

The use of 18F-DCFPyL allowed for a model of large-scale centralized production and distribution (20), which was successful for the most part with meeting demand. Though it led to ease of production and cost savings, this model was not without challenges. In particular, the Thunder Bay site in Northern Ontario faced logistical challenges due to the impact of the COVID-19 pandemic on commercial air travel and transport of the radiopharmaceutical agents. An advantage of local production of radiopharmaceuticals, whether by a local cyclotron or generator, is that it could lead to more stable and reliable delivery by removing the uncertainties of transport logistics and potentially serving as a backup redundant source in the event of production issues at other facilities. A center in Italy faced with the challenge of not having an on-site cyclotron demonstrated that it was feasible to synthesize 18F-PSMA-1007 from 18F- imported from different external suppliers (21). However in the case of Ontario, the absence of local cyclotrons was not the reason for a centralized production model. This model was also a requirement due to existing licensing and regulatory approvals. Licensing for 18F-DCFPyL was held by the CPDC (Centre for Probe Development and Commercialization), which had Health Canada approvals for production at the Toronto CanProbe facility (Canadian Molecular Probe Consortium, a joint venture between the University Health Network (UHN) and the CPDC). Given the complexities and costs of licensing requirements and clinical use approvals, there continues to be regulatory barriers in the way of local production despite the availability of a cyclotron in Thunder Bay. Whether centralized or decentralized production best fits the geography and needs of a jurisdiction is an important question to be considered when deciding between 68Ga and 18F-based radiopharmaceuticals.

Challenges aside, stakeholder feedback was overall positive regarding the impact of PSMA PET/CT on the care of men enrolled on the Registry. The rates of detection and management change in the Registry were consistent with the experiences in other jurisdictions (5, 6, 22) and stakeholder feedback affirmed the clinical value of PSMA PET/CT studies. Additionally, the Registry consent provides for data linkage to other provincial administrative databases, providing opportunities to explore other downstream care impacts of PSMA PET/CT such as patterns of salvage radiotherapy utilization for biochemical failure post radical prostatectomy, as well as developing predictive models to improve the pre-test probability of an informative PSMA PET/CT to encourage appropriate utilization. Finally, the Registry study is providing a valuable opportunity for nuclear medicine physicians throughout the Province to gain experience with this new PET imaging modality. Existing peer to peer networks are being leveraged among nuclear medicine physicians for knowledge dissemination and shortening of learning curves.

### Future Directions

As the PREP registry study further accrues patients, we hope to understand and build evidence on the most appropriate and impactful indications for PSMA PET. Currently, there is strong global evidence supporting the use of PSMA PET in the biochemical recurrent setting (23). The evidence for PSMA PET in other indications is not as clear (12). Through PREP, we seek to continue to assess the use of PSMA PET in other indications, for example, in primary staging of medium or high-risk prostate cancer or in primary detection of tumor in complex cases where there exists clinical suspicion for prostate cancer despite a negative conventional workup, including multiparametric prostate MRI and systematic biopsies. PREP includes an adjudicated “decision making” access cohort as it is acknowledged that patients outside of the PREP defined cohorts (**Table 1**) may also benefit from PSMA PET informed decision making.

Though the PREP registry has provided many men in Ontario with the access to PSMA PET scans, the ultimate goal is to build adequately robust evidence for Health Canada approval and provincial funding for the appropriate indications. Likely, the first indication to gain Health Canada approval will be men with biochemically recurrent prostate cancer – once that happens, OH-CCO can transition this aspect of the registry into a funded clinical service. However, support for additional indications will require prospective data demonstrating improved clinical outcomes from PET-directed therapy. Such randomized trials are beginning to read out (24); for example, ongoing trials in Ontario are evaluating metastasis directed and PET-guided treatments in recurrent and high risk prostate cancer. Of note, the Canadian PATRON (NCT04557501) trial is assessing whether PSMA PET-guided intensification of therapy would improve clinical outcomes compared to the current standard of care, in both the biochemical recurrence and primary staging settings. As the clinical evidence base supporting the use of PSMA PET/CT develops, building distributed provincial radiopharmaceutical production and PET/CT scanning capacity to meet new indications will be necessary to meet future need and ensure equitable access province wide.

## Conclusion

In summary, the PREP registry study was launched in Ontario in 2018 as a pragmatic response to enable access to PSMA PET/CT imaging on a provincial scale, to build evidence and inform appropriate indications for PSMA PET, and to support the nuclear medicine community in gaining experience with the novel 18F-DCFPyL PSMA radiopharmaceutical. Through key stakeholder interviews, we elicited the successes, barriers and logistics of developing a provincial registry, including the challenges of radiopharmaceutical production and distribution, funding models and the impact of the pandemic. We share these results for other provinces and countries seeking to improve access to novel PET imaging for their patients. Overall, we demonstrate that the PREP registry has been a successful endeavor in providing access and real-world experience of a promising advanced prostate cancer imaging modality in Canada. Many of the lessons learned from this registry may be applicable to the introduction of novel radiopharmaceuticals in other jurisdictions.

## Data Availability Statement

The data that support the findings of this study are available from the corresponding author (GB) upon reasonable request.

## Ethics Statement

Health Canada CTA approval obtained for clinical use of the PSMA PET/CT radiotracer. Clinical protocol approved by the Ontario Cancer Research Ethics Board (OCREB ID 1398).

## Author Contributions

GB and UM conceived and designed the Registry Study. SY and GS designed interview guides, conducted key stakeholder interviews and engaged in qualitative data analysis. The manuscript was drafted by SY, GB, and UM and edited by all authors. All authors contributed to the article and approved the submitted version.

## Funding

The PREP Registry is funded by Cancer Care Ontario, an agency of the Ontario Ministry of Health.

## Conflict of Interest

UM is a consultant for POINT Biopharma Inc. SY holds common shares in Lantheus Holdings Inc.

The remaining authors declare that the research was conducted in the absence of any commercial or financial relationships that could be construed as a potential conflict of interest.

## Publisher’s Note

All claims expressed in this article are solely those of the authors and do not necessarily represent those of their affiliated organizations, or those of the publisher, the editors and the reviewers. Any product that may be evaluated in this article, or claim that may be made by its manufacturer, is not guaranteed or endorsed by the publisher.
