# Supplementary material for: Establishing a Provincial Registry for Recurrent Prostate Cancer: Providing Access to PSMA PET/CT in Ontario, Canada
Source: Front Oncol. 2021 Aug 2;11:722430. doi: 10.3389/fonc.2021.722430 (PMC8366560; doi:10.3389/fonc.2021.722430)
Supplement: Supplementary file 1 [file DataSheet_1.zip › Supplementary material.pdf]

PSMA-PET Registry for Recurrent Prostate Cancer (PREP)

Date: April 16, 2020

Funder: Cancer Care Ontario

Overall Co-Principal Investigators
Glenn Bauman, MD
Ur Metser, MD
Antonio Finelli, MD

Site: London Health Sciences Centre
Oncology: Glenn Bauman, MD
Imaging: Irina Rachinsky, MD
Urology: Joseph Chin, MD*

Site: Princess Margaret Cancer Centre, UHN
Imaging: Ur Metser, MD
Urology: Antonio Finelli, MD*

Site: Toronto Sunnybrook Cancer Centre
Imaging: Robert Wolfson, MD
Urology: Laurence Klotz, MD*

Site: St. Joseph's Healthcare Hamilton
Imaging: Katherine Zukotynski, MD
Urology: Anil Kapoor, MD*
Bobby Shayegan, MD

Site: Ottawa Regional Cancer Centre
Imaging: Eugene Leung, MD
Urology: Luke Lavallee, MD*
Chris Morash, MD

Site: Thunder Bay Regional Cancer Centre
Oncology: Marlon Hagerty, MD*
Imaging: Jonathan Boekhoud, MD
Urology: Walid Shahrour, MD

*Site Primary Investigator/Contact

Title: PSMA-PET Registry for Recurrent Prostate Cancer (PREP)

GOAL:

To institute a province-wide registry leveraging the availability of a new Positron Emission Tomography tracer, 18F-DCFPyL and PET expertise across Ontario centers to improve our ability to characterize patterns of recurrence and personalize therapies in men with recurrent prostate cancer after prostatectomy or primary radiotherapy.

DESIGN:

Prospective Provincial Registry

BACKGROUND

While radiotherapy or prostatectomy are curative treatment for many men with localized prostate cancer, the rate of biochemical failure after primary treatment ranges from 20-40% depending on pre-treatment characteristics (PSA) and post-treatment stage (pT and pN stages). In particular men with node positive disease or with persistently detectable PSA at early time points (<6 months) post radiotherapy are felt to be at high risk of relapse. Similarly rates of biochemical failure after adjuvant or salvage prostate bed radiotherapy following prostatectomy range from 20-60%. Those men with biochemical progression despite prior intensive pelvic radiotherapy (prostatectomy +/- pelvic radiotherapy) and salvage hormone therapy are at high risk of developing symptomatic metastatic disease and can be identified by a rising PSA in the presence of a castrate testosterone levels. Men treated with primary radiotherapy have approximately 50% chance of successful local salvage therapy (no matter what local salvage modality is used) at the time of biochemical failure, suggesting unrecognized extra-prostatic/metastatic disease is a limiting factor in the management of this group of men. In each of these situations, men with suspected persistent or recurrent disease can be identified on the basis of biochemical or pathologic factors, however the inability to localize sites of disease hampers the ability to personalize therapies. For example, in the absence of spatial information about sites of disease, men may be managed in a palliative fashion with observation or hormone/systemic therapy only, or may receive ineffective local salvage (for example prostate bed radiotherapy) or may forgo a chance for lesion directed therapy that might delay the need for salvage hormone/systemic therapy (through surgery or stereotactic radiotherapy to treat isolated metastatic sites). In the face of this uncertainty about metastatic sites of recurrence those men with less favorable features at the time of salvage may be treated with either surveillance or androgen deprivation therapy with local salvage offered less frequently.(1) Such approaches (hormones or observation) are palliative in intent and may deprive some men of potentially curative salvage treatments, may result in morbidity at the time of clinical failure or expose men prematurely to the significant side effects (such as constitutional symptoms, metabolic syndrome, osteoporosis among others with hormone therapy). (2) (3) This situation is compounded in men who already have been treated with adjuvant or salvage radiotherapy where an inability to identify focally recurrent disease using current imaging drives management with either surveillance or hormone therapy, or those men with progression on hormone therapy where selection for salvage secondary hormone therapy or chemotherapy is based on development of symptomatic metastatic disease. For those men with suspected local recurrence after primary radiotherapy, salvage local therapies carry significant morbidity so better patient selection for those men who stand to benefit most from those local salvage treatments is needed. For those men treated for oligometastatic disease, subsequent oliogmetastatic recurrences can be observed, necessitating the need to identify men who may be suitable for ongoing lesion directed therapy versus those men who should be converted to systemic therapies because of extensive progression. Finally, given the variable course and multiple treatment options for men with prostate cancer, there are other individual scenarios where accurate staging could influence treatment selection, for example suspected local failure after non-conventional local or focal ablative therapies like high intensity focused ultrasound or laser ablation.

Metastatic prostate cancer and the oligometastatic state

Traditionally, the identification of extraprostatic disease (typically nodal or bone) has been regarded as a marker of widespread metastatic disease with a subsequent palliative approach with either expectant management or hormone therapy (more recently hormone therapy and chemotherapy for men with large volume metastatic disease). That said, autopsy series suggest that some men may have limited metastatic burden and other series have demonstrated that the number of metastatic sites is correlated with prostate cancer specific mortality and response

to hormone therapy with men with a low burden of disease (<3-5 sites of metastases) enjoying longer survival. For other disease sites such as colorectal cancer, the concept of the "oligometastatic state" has been accepted. Such patients demonstrate limited sites of metastatic disease and selected patients appear to benefit from aggressive treatment of such oligometastatic sites through surgery or other ablative therapies such as stereotactic body radiotherapy (SBRT/SABR). (4) The concept of the "oligometastatic state" remains controversial in prostate cancer although treatment paradigms including intensified ablative therapies to limited sites of metastatic disease are emerging. (5-8) Such treatments may be delivered with either the intent of augmenting response to traditional salvage with hormone therapy or with the intent of limiting or deferring the use of hormone therapy to avoid the potential toxicities of this treatment. The appropriate indications for the utilization of stereotactic radiotherapy for oligometastatic disease are poorly defined for patients with prostate cancer although institutional reports of successful salvage with hypofractionated SBRT for local, nodal and bone recurrence have been described. In these series, high rates of local control for treated lesions are noted along with low rates of toxicity (Table 1). For those men where local salvage treatments are considered, uncertainty in restaging may expose men to the side effects of those treatments without benefit (5). Freedom from biochemical or clinical progression is noted in 30-50% over time periods of 1-3 years in a recent systematic review (7), however heterogeneity in patient series (differences in use of hormone therapy and repeat salvage therapy make comparisons difficult). (5) (9) (6) As stated in the meta-analysis "for MDT [metastases directed treatment] to be successful, three main prerequisites should be fulfilled: **(1) accurate imaging to detect early metastases**, (2) complete eradication of all oligometastatic sites, and (3) acceptable toxicity". (7)

Restaging prior to salvage therapies

As outlined above, there are a number of lesion-targeted therapies for potential deployment for men with locally recurrent or limited metastatic disease after primary or adjuvant/salvage radiotherapy. A key barrier to the further development of these strategies is limitation in our ability to identify sites of recurrence and burden of disease at the time of early biochemical failure post radiotherapy. There is a need to develop better ways to stage men with recurrence post radiotherapy and treat men with isolated locally recurrent and potentially those with oligometastatic disease with the goal of safely deferring (and potentially in some men avoiding) the institution of androgen deprivation. (10)

To this end, newer imaging technologies have been explored. For men with suspected local recurrence, the availability of multi-parametric MRI has been useful in identifying prostate bed foci suspicious for recurrence that can then serve as a target for confirmatory biopsy and/or salvage RT. Pelvic MRI however remains relatively insensitive to detecting extra-prostatic disease such as nodal failure. The development of whole body MRI techniques has allowed the use of multi-parametric MRI as a screening tool for systemic disease with whole body diffusion MRI showing particular promise. (11) However, whole body techniques are not yet widely available.

In the last 10 years, the use of new PET tracers for the systemic staging of prostate cancer has been described. In particular, small peptide tracers directed against Prostate Specific Membrane Antigen (PSMA) and radiolabeled choline analogs (11C-Choline, 18F-Fluorocholine) have been introduced and demonstrated the ability to detect bone and nodal disease not identified by conventional imaging (bone scan or CT). (12, 13) The comparison of these agents against each other and standard of care imaging has not been conducted in a comprehensive fashion although a recent retrospective comparison suggested PSMA based PET probes may be superior to FCH in the characterization of metastatic disease. (14) In a prospective study, 38 men with rising PSA following initial radical treatment were evaluated with a PSMA PET probe, Glu-NH-CO-NH-Lys-(Ahx)-[68Ga(HBED-CC)](PSMA) and 18F-Fluoromethylcholine (FMC). Imaging with PET/CT plus diagnostic CT were obtained. Among this cohort, 34/38 (89%) were post radical prostatectomy (RP), 4/38 (11%) were post radiotherapy (RT) and 12/38 (32%) both post RP and RT. Mean PSA was 1.74 ± 2.54 ng/ml. and 68% (26/38) had a positive scan. Of these, 14/26 (54%) were positive on PSMA alone, and 42% (11/26) on both FMC and PSMA, and 1/26 on FMC alone (false positive on biopsy). All biopsied PSMA lesions were true positive (6/6). Diagnostic CT was inconclusive in all men. With PSA <0.5ng/ml, PSMA detection was 50% vs. 12.5% for FMC for PSA 0.5-2.0 ng/ml, 69% PSMA vs. 31% for FMC, and PSA >2.0, 86% PSMA vs. 57% FMC. There was a 63% (24/38) management impact of PET imaging. It was concluded that PSMA demonstrated a significantly higher detection rate with a high overall management impact.(15) The results of this study suggest a high prevalence of lesions outside of the prostate bed/prostate at the time of biochemical recurrence. These results may be

influenced by the fact that the PSA results at the time of imaging was relatively high, given most patients imaged (89%) had a prior RP. Likewise, Albisinni et al noted a management impact in 76% of men imaged with 68Ga-PSMA amongst 131 men imaged for biochemical failure after prostatectomy. They noted high (>75%) lesion detection rate among men with a PSA>0.5ng/ml and changes in treatment included decisions to defer radiotherapy or hormone therapy, lesion directed treatment with stereotactic RT, salvage prostate bed RT or regional node salvage with RT or surgery. (16) In a small study (n=14) comparing a 68-Ga based PSMA probe against an 18-F based PSMA probe in men with recurrence post prostatectomy or radiotherapy, improved performance for the 18-F probe was noted (higher SUV and better sensitivity for lesion detection) with improved detection of bone, nodal and intra-prostatic recurrences with 18F- PSMA. Thus 18-F PSMA PET imaging appears to be a promising new modality compared to other agents (FDG, FCH) and other PSMA probes such as 68-Ga PSMA. (17)

Study rationale

In Ontario, there is a track record of evaluation of new PET imaging indications in prospective clinical trials(18), capabilities for novel probe development(19) as well as a track record of locally targeted salvage prostate therapies and SBRT capabilities(20) for the treatment of oligometastatic disease. (21-23) Integrated PET/CT scanners are available across the province and are in routine use for cancer staging and re-staging under the Ontario PET access program. Ongoing multi-institutional prospective trials of the use of [18F]DCFPyL PET/CT are underway in Ontario [NCT02856100, NCT02793284] and are examining the use of PET/CT for restaging men at the time of biochemical failure after primary radiotherapy or to monitor response to therapy in metastatic castrate resistant prostate cancer. In addition there have been recently completed prospective trials of staging and restaging studies using 18F-Fluorocholine for men with high risk disease and initial biochemical failure after RP (NCT01993160, NCT02131649). Awareness of availability of PET/CT in these trials, plus increasing reports of the use of staging PET/CT and lesion directed therapy (radiotherapy and surgery) for oligometastatic disease and a highly motivated patient population who are seeking alternatives to conservative approaches to recurrent disease (observation or hormone therapy) have led to increased demand for access to PET/CT in the setting of recurrent disease. Such demand is manifest in direct communication to study investigators conducting ongoing protocols as well as requests for access to PET/CT through the Out of Country Program through Cancer Care Ontario. The proposed registry seeks to exploit the existing provincial capabilities to provide PET/CT imaging for recurrent prostate cancer and in doing so characterize patterns of recurrence, salvage therapies and clinical outcomes in men with recurrent disease after radical prostatectomy, primary radiotherapy and other emerging non-conventional local and focal therapies. A prospective registry will also help to define feasibility and utility of using new imaging for identifying men with limited sites of recurrent prostate cancer after primary or adjuvant/salvage radiotherapy following prostatectomy. Specifically, the PET agent to be investigated is a 18F labeled small molecule PSMA probe, [18F]DCFPyL(24), that has shown promise in preclinical and clinical studies and is in production through the Centre for Probe Development and Commercialization (CPDC, Hamilton, Ontario). (19)

We will investigate the ability of [18F]DCFPyL PET/CT imaging ("**PSMA PET/CT**") to detect sites of recurrence and guide management in several scenarios of suspected persistent or recurrent disease post radical-prostatectomy at various decision points in the disease trajectory as well as in men with biochemical failure after primary radiotherapy. An additional access cohort will also be included to capture other potential indications where [18F]DCFPyL PET/CT could potentially influence patient management. The specific cohorts to be explored are as follows:

Cohort 1. Post prostatectomy node positive disease or persistently detectable PSA

Patients who have node positive disease or persistently detectable PSA after prostatectomy are recognized to have an increased risk of subsequent metastatic failure. At the same time, the optimal management of these men remains undetermined with observation, adjuvant hormone therapy, adjuvant pelvic radiotherapy or adjuvant radiotherapy and hormone therapy considered possible options. (25) Improved staging of men with surgically confirmed node positive disease could help inform the selection of adjuvant therapies by identifying those men with occult metastatic disease (who may be suitable for adjuvant hormone therapy), men with additional pelvic nodal disease not resected by standard lymphadenectomy (who may be suitable for adjuvant pelvic radiotherapy) or those with no obvious disease (who may be suitable for observation).

PSMA PET result	Possible PSMA PET/CT informed management
Negative or limited sites of disease	Observation
Uptake in prostate bed or pelvic nodes only	Add pelvic radiotherapy
Uptake in pelvic nodes only	Hormone Therapy
Limited extra-pelvic disease	Metastasectomy
Limited extra-pelvic disease	Stereotactic ablative RT
Extra pelvic disease	Hormone Therapy
Extensive pelvic disease	Chemotherapy

Cohort 2. Biochemical failure post prostatectomy
Following prostatectomy a persistently rising PSA is a sensitive indicator of recurrent disease. Such recurrence may be limited to the prostate bed (potentially treatable with salvage prostate bed radiotherapy), pelvic regional lymph nodes (potentially treatable with radiation, hormone therapy or combinations) or extra pelvic recurrence (potentially treatable by lesion directed therapy like surgery or radiotherapy, hormone therapy or combinations). (25)

PSMA PET result	Possible PSMA PET/CT informed management
Negative or limited sites of disease	Observation
Uptake in prostate bed or pelvic nodes only	Pelvic radiotherapy
Uptake in pelvic nodes only	Add pelvic node dissection
Uptake in pelvic nodes only	Hormone Therapy
Limited extra-pelvic disease	Metastasectomy
Limited extra-pelvic disease	Stereotactic ablative RT
Extra pelvic disease	Hormone Therapy
Extensive pelvic disease	Chemotherapy

Cohort 3. Failure following radical prostatectomy followed by adjuvant or salvage prostate bed radiotherapy
While the indications for adjuvant radiotherapy following prostatectomy are reasonably well defined (positive margins, pT3 disease) and randomized trials have demonstrated a decrease in biochemical failure by approximately 50% in men receiving adjuvant therapy, not all men are offered or accept adjuvant radiotherapy because of concerns regarding potential toxicity. In men who receive adjuvant therapy subsequent biochemical failure may still be seen in up to 20%. In those men who receive salvage radiotherapy after prostatectomy for biochemical failure, successful treatment results in approximately 50% with the other half going on to a second biochemical failure. Thus, there is a substantial population of men who may experience biochemical failure despite intensive local treatment with prostatectomy plus prostate bed radiotherapy. In these men salvage hormonal therapy or observation are the typical options with many men opting for observation because of concerns regarding toxicity of hormone therapy. A recent study of 11C-choline suggested that among this population regional nodal recurrence may be the most frequent site of recurrence. (26) Confirmation of such regional recurrence could allow regional salvage with radiotherapy or lymph node dissection. Likewise limited extra pelvic disease might be treated with lesion directed therapy. (25)

PSMA PET result	Possible PSMA PET/CT informed management
Negative or limited sites of disease	Observation
Uptake in pelvic nodes only	Add pelvic node dissection
Uptake in pelvic nodes only	Add pelvic node radiotherapy
Uptake in pelvic nodes only	Hormone Therapy

Limited extra-pelvic disease	Metastasectomy
Limited extra-pelvic disease	Stereotactic ablative RT
Extra pelvic disease	Hormone Therapy
Extensive pelvic disease	Chemotherapy

Cohort 4. Rising PSA post prostatectomy or radiotherapy while on hormone therapy
After prior local pelvic therapy with prostatectomy or radiotherapy many men will opt for salvage hormonal therapy at the time of subsequent biochemical failure. In addition, some men with high risk disease at diagnosis will receive adjuvant hormone therapy for 2-3 years following local therapy. Such salvage or adjuvant treatment may be successful for a time but subsequent rise while on hormone therapy is typically an indication of subclinical metastatic disease. Salvage systemic therapies include chemotherapy and second line hormone therapies such as enzalutamide or abiraterone, however such therapies are expensive and more toxic and physicians and patients are reluctant to undertake such therapies unless the patient is symptomatic or there is demonstrated metastatic disease. Early detection of sites of castrate resistant disease may provide an opportunity for lesion directed therapy such as stereotactic radiotherapy and or may help inform decisions to institute salvage systemic therapy. (25)

**PSMA PET result**	**Possible PSMA PET/CT informed management**
Negative or limited sites of disease	Observation
Uptake in pelvic nodes only	Add pelvic node dissection
Uptake in pelvic nodes only	Add pelvic node radiotherapy
Limited extra-pelvic disease	Metastasectomy
Limited extra-pelvic disease	Stereotactic ablative RT
Extra pelvic disease	Second line Hormone Therapy
Extensive pelvic disease	Chemotherapy

Cohort 5. Evaluation of PSMA directed treatment
Ultimately, the usefulness of PSMA PET will be in its ability to influence treatment decisions. For example, better characterization of location and volume of prostate cancer recurrence will allow the more rational selection of targeted therapies (such as local prostate or prostate bed treatment, use of regional nodal irradiation or salvage surgery or optimization of systemic therapies). Such an approach has been demonstrated to allow the successful deferral of androgen deprivation therapy or the use of short course androgen deprivation therapy as an alternative to prolonged therapy in men with oligometastatic disease (STOMP and ORIOLE trials). Experience with this approach suggests some men over time will re-present with new sites of oligometastatic disease and be successfully salvaged with further lesion directed therapy. In addition, response to systemic therapy in the setting of hormone naive disease has been correlated with volume of disease when conventional imaging is used but volume correlations with PET based staging have not been characterized. While PSA response may provide a global assessment of disease response, in some cases (i.e. androgen suppression) PSA response may not necessarily reflect disease response. Repeat PSMA PET imaging in this cohort will help further characterize the response of patients who have had a change in treatment based on PSMA PET imaging. (25)

**PSMA PET result**	**Possible PSMA PET/CT informed management**
Negative or limited sites of disease	Observation
Uptake in pelvic nodes only	Add pelvic node dissection
Uptake in pelvic nodes only	Add pelvic node radiotherapy
Limited extra-pelvic disease	Metastasectomy
Limited extra-pelvic disease	Stereotactic ablative RT
Extra pelvic disease	Second line Hormone Therapy

Extensive pelvic disease	Chemotherapy

Cohort 6. While many men receive prostatectomy as their primary therapy for prostate cancer, radiotherapy (external beam radiotherapy, brachytherapy or combinations together or with ADT therapy) is the primary treatment for men either unfit for surgery or for men who wish to avoid an invasive procedure or the potential side effects of surgery. In men receiving primary radiotherapy, biochemical failure may be seen in up to 25-30% of men. Particularly in those men with localized disease at initial treatment, accurate restaging may facilitate the selection of men for local salvage therapy such as salvage prostatectomy or other local salvage interventions such as cryotherapy, high intensity focused ultrasound or repeat radiotherapy (typically brachytherapy). The prospective PICs study (NCT02793284), evaluated 18F-DCFPyL in the radio-recurrent setting and demonstrated the ability to detect extra-prostatic recurrence in approximately twice as many men (38% vs. 18%) compared to conventional imaging) and changed management based on conventional imaging in approximately half of men (personal communication). This cohort will evaluate 18F-DCFPyL to provide further validation of its performance in this patient population and provide continued access for this patient population now that the PICs study has concluded.

PSMA PET result	Possible PSMA PET/CT informed management
Negative or limited sites of disease	Observation
Uptake in prostate only	Local salvage
Uptake in pelvic nodes only	Add pelvic node dissection or nodal radiotherapy
Uptake in pelvic nodes only	Hormone Therapy
Limited extra-pelvic disease	Metastasectomy
Limited extra-pelvic disease	Stereotactic ablative RT
Extra pelvic disease	Hormone Therapy
Extensive pelvic disease	Chemotherapy

Cohort 7. [18F]DCFPyL as a problem-solving tool in patients with prostate cancer when confirmation of site of disease and/or disease extent may impact clinical management over and above the information provided by conventional imaging. All requests for inclusion in the Registry under Cohort 7 will be adjudicated by an expert panel at Cancer Care Ontario. The panel will consist of 3 physicians, including oncologists with prostate cancer expertise and radiologists/ nuclear medicine physicians with PET expertise. The panel will receive a summary of the patient's relevant clinical history, along with supporting documents. The panel will determine whether findings on PET may impact patient management. Decisions will be made by a majority. If the panel cannot reach a decision, input from a second panel of experts may be requested. The final decision of the expert panel will be shared with the study sponsor. In cases where PET is not approved by the panel, referring physician may appeal the decision after providing further clinical information or further explanation on how PET would impact patient management.

HYPOTHESIS:

In the setting of men treated with initial prostatectomy or primary radiotherapy experiencing biochemical failure on current management or have high risk disease at the time of initial surgery (node positive or persistently detectable PSA within 3 months post-surgery), PSMA PET will be able to detect men with limited number of sites of recurrent disease (4 or less) who can potentially be treated with metastases directed therapy with the intent of deferring or avoiding the need for systemic therapy interventions.

OBJECTIVES

Primary Objective PREP

To determine the proportion of men with sites of disease identified at the time of early biochemical failure in high risk disease in men treated with prostatectomy with or without adjuvant or salvage pelvic radiotherapy or hormone therapy as well as men treated with primary radiotherapy. This will be calculated as the number of men with at least 1 PSMA PET detected lesion that is scored as +5 (definitely malignant) or +4 (probably malignant) according to the local imaging read.

Primary Objective PREP Phase 2
To determine the proportion of men with sites of disease identified at the time of early biochemical failure in high risk disease in men treated with prostatectomy with or without adjuvant or salvage pelvic radiotherapy or hormone therapy as well as men treated with primary radiotherapy when PSMA PET/CT is used without prescreening with conventional imaging. This will be calculated as the number of men with at least 1 PSMA PET detected lesion that is scored as +5 (definitely malignant) or +4 (probably malignant) according to the local imaging read.

Secondary Objectives
1. Determine correlations between PSA levels at time of imaging and presence of disease detected on PSMA PET
2. Determine the proportion of men with oligometastatic recurrence (four or fewer sites including the prostate bed if positive) detected on PSMA PET as calculated by the number of men with 4 or fewer lesions identified as +5 (definitely malignant) or +4 (probably malignant) divided by the number of men with any number of +5 or +4 lesions detected.
3. Determine the number of men who have a change in management as indicated by responses from referring physicians on an impact questionnaire completed after PSMA PET scans are reported.
4. Determine the actual management delivered within 6 months by linkage to existing health information registries:
    a. Delivery of radiotherapy (anatomic site, dose and fractionation) – Cancer Care Ontario
    b. Biopsy of suspected recurrences (anatomic site, histology) – Provincial pathology database
    c. Use of salvage lymph node dissections – CIHI
    d. Use of salvage hormonal therapy/androgen deprivation
5. Compare PSA response at 6 months against PSA at the time of PSMA PET obtained through linkage to the Ontario Laboratory Information Services and correlate with actual management as determined in 4.
6. To compare the detection rates of PSMA PET/CT when conventional imaging is used as part of eligibility criteria (PREP) versus when conventional imaging is omitted (PREP Phase 2)

STUDY DESIGN
The study will be conducted as a prospective registry. Seven cohorts of men will be examined: 1) men who are node positive or who have a persistently detectable PSA after initial radical prostatectomy, 2) men with biochemical failure after initial RP, 3) biochemical failure after initial RP and salvage RT, and 4) biochemical failure after initial RP with or without adjuvant/salvage RT who are currently on salvage hormone therapy, 5) men who have prior metastases directed treatment for oligometastatic disease with subsequent biochemical failure, 6) men with a rising PSA (Phoenix Criteria) following primary radiotherapy, 7) men whose clinical scenarios fall outside of cohorts 1-6 but where an independent adjudication process determines PSMA PET could provide clinically meaningful information. Participating centres will be responsible for submitting patient demographic data to establish eligibility upon review by the central PET registry, obtaining patient consent including consent for linkage of their data to existing provincial patient registries and providing results of the [18F]DCFPyL PET/CT on a standardized reporting form. Other patient related outcomes (interventions post PET/CT) will be assessed by linkage to existing patient registries to determine use of salvage surgical and radiotherapy procedures and use of hormone and systemic therapy. This registry study is expected to take 4 years to complete with an additional one year of follow-up to capture patient outcomes during which time additional patient enrollment on the individual cohorts will continue.

STUDY DESIGN – PHASE 2 REVISION
During Phase I of the PREP protocol, all patients were required to have conventional imaging (computed tomography and bone scan) either negative or demonstrating oligometastatic disease. Based on an analysis of the first 400 patients accrued, the majority of patients (76%) had negative conventional imaging, with 24% had oligometastatic disease. Overall PSMA PET/CT detection rates of new lesions were similar among patients with negative conventional imaging (59%) versus oligometastatic disease on conventional imaging (63%). There were 12% of patients who had extensive disease on subsequent PSMA PET/CT with the rest of patients having negative scans or oligometastatic disease. Of these patients half (6%) had negative conventional imaging and half had oligometastatic

disease on conventional imaging (patients who had extensive metastatic disease on conventional imaging were not eligible for participation in PREP). Given these findings, it would appear that conventional imaging is not providing additional value in terms of overall staging compared to PSMA PET/CT alone, although this conclusion is limited somewhat by the fact that patients with extensive disease were excluded from participation from the first Phase of PSMA PREP. Given the historical data suggesting low rates of positive conventional imaging at PSA levels (<10 ng/ml) where actionable (locally recurrent or oligometastatic) disease is most likely to be found(27, 28) it is reasonable to hypothesize that omission of conventional imaging as criteria for PSMA PET/CT should not materially affect the performance of the test. Omission of conventional imaging would save costs and also improve the efficiency of evaluating men as currently waits for conventional imaging can add weeks to months to the waiting time for accessing PSMA PET/CT as part of PREP. To investigate the hypothesis that conventional imaging is not adding to the information provided by PSMA PET/CT alone, PREP Phase 2 will retain the same study design as Phase I but will remove bone scan and computed tomography as criteria for entry into the study except for those patients with higher PSA (>10 ng/ml). Identical cohort sizes will be accrued in Phase 2 to permit comparison of detection rates with similar confidence intervals with and without conventional imaging. Transition to PREP Phase 2 will occur when overall accrual to PREP has exceeded 80% of target.

ELIGIBILITY

Eligibility status must be confirmed by the local investigator or designate and will be confirmed by the provincial PET registry prior to enrollment. It is important that no exceptions be made to the eligibility criteria.

Inclusion Criteria

1. Written informed consent obtained
2. Male, Age ≥ 18 years
3. Prior primary treatment for prostate cancer with curative intent such as radical prostatectomy **or** radiotherapy for localized prostate cancer. Unless PET/CT requested as part of Cohort 7.
4. Suspected persistent or recurrent disease defined as one of the following:
    a. High risk disease at the time of radical prostatectomy characterized by pathologically involved node(s) **or** persistently detectable PSA (>0.1ng/ml) within 3 months post-surgery
    b. Primary treatment for prostate cancer and biochemical failure (BF) with current management according to the following:
        i. Following primary radical prostatectomy, BF is defined as rising PSA on at least 2 occasions measured at least 1 month apart and with the most recent PSA measured at >0.1 ng/ml
        ii. Following primary radiotherapy for localized disease, BF is defined according to the Phoenix Definition, which is rising PSA on at least 2 occasions measured at least 1 month apart and with the most recent PSA measured greater than the nadir PSA + 2.0 ng/ml.

    Unless PET/CT requested as part of Cohort 7.
5. Patient scenario falls into one of the 7 pre-defined cohorts. When patient scenario falls outside cohorts 1-6 participation in the Registry must be approved through the established CCO adjudication process for cohort 7.
6. Conventional imaging consisting of bone scan and abdo-pelvic CT scan within 3 months of registration that is either equivocal, negative (no lesions) or positive for oligometastatic disease (4 or fewer unequivocal lesions identified). Registration is defined as Form A: Eligibility is complete.
7. Karnofsky performance status 70 or better (ECOG 0, 1)

Exclusion Criteria

1. Prostate cancer with significant sarcomatoid or spindle cell or neuroendocrine small cell components
2. Extensive metastatic disease on conventional restaging (>4 sites unequivocally positive for disease). Unless PET/CT requested as part of Cohort 7.
3. Prior PSMA PET scan within 6 months of enrollment
4. Patient cannot lie still for at least 60 minutes or comply with imaging
5. Patients falling outside of Cohorts 1-6 where independent adjudication by CCO does not support participation in the Registry

ELIGIBILITY – PREP PHASE 2

Eligibility status must be confirmed by the local investigator or designate and will be confirmed by the provincial PET registry prior to enrollment. It is important that no exceptions be made to the eligibility criteria.

Inclusion Criteria

1. Written informed consent obtained
2. Male, Age ≥ 18 years
3. Prior primary treatment for prostate cancer with curative intent such as radical prostatectomy **or** radiotherapy for localized prostate cancer. Unless PET/CT requested as part of Cohort 7.
4. Suspected persistent or recurrent disease defined as one of the following:
    a. High risk disease at the time of radical prostatectomy characterized by pathologically involved node(s) **or** persistently detectable PSA (>0.1ng/ml) within 3 months post-surgery
    b. Primary treatment for prostate cancer and biochemical failure (BF) with current management according to the following:
        i. Following primary radical prostatectomy, BF is defined as rising PSA on at least 2 occasions measured at least 1 month apart and with the most recent PSA measured at >0.1 ng/ml
        ii. Following primary radiotherapy for localized disease, BF is defined according to the Phoenix Definition, which is rising PSA on at least 2 occasions measured at least 1 month apart and with the most recent PSA measured greater than the nadir PSA + 2.0 ng/ml.

    Unless PET/CT requested as part of Cohort 7.
5. Patient scenario falls into one of the 7 pre-defined cohorts. When patient scenario falls outside cohorts 1-6 participation in the Registry must be approved through the established CCO adjudication process for cohort 7.
6. Karnofsky performance status 70 or better (ECOG 0, 1)
7. If PSA >10 ng/ml, conventional imaging consisting of bone scan and abdominal-pelvic CT scan performed within 3 months of <u>registration</u> that is either equivocal, negative (no lesions) or positive for oligometastatic disease (4 or fewer unequivocal lesions identified). <u>Registration</u> is defined as Form A: Eligibility is complete.

Exclusion Criteria

1. Prostate cancer with significant sarcomatoid or spindle cell or neuroendocrine small cell components
2. Prior PSMA PET scan within 6 months of enrollment
3. Patient cannot lie still for at least 60 minutes or comply with imaging
4. Patients falling outside of Cohorts 1-6 where independent adjudication by CCO does not support participation in the Registry

Assessment of lesion detection rate

PSMA PET/CT will be interpreted by local readers. Participating sites will be required to submit a standardized PSMA PET/CT results report. Those patients with four or fewer sites of disease identified on PSMA PET/CT will be classified as "oligometastatic".

Assessment of PSMA PET/CT informed management

As part of the patient eligibility for PSMA PET/CT referring physicians will complete a PSMA PET Management Impact Worksheet after the PSMA PET/CT information is provided. The questionnaire will ask the physicians to indicate if there was a change in management based on the results of the PSMA PET/CT. Actual interventions following completion of the PSMA PET/CT will be tracked by linkage to provincial registries.

STUDY PROCEDURES

Screening visit

Urologists and Oncologists will identify potentially eligible patients and will be responsible for obtaining consent and completing pre-PET registration materials. Patient consent forms will be held locally as part of the patient's medical record.

Patient Approval for PSMA PET/CT

Registration materials for the requested PSMA PET as part of the registry will be sent to the local PET Centre participating in the registry and reviewed by the local Imaging investigator. Provided all eligibility criteria are met and pre-imaging questionnaires are completed the PSMA PET scan will be scheduled within 6 months of registration. Cancer Care Ontario will also receive registration materials for the purposes of tracking patients on the registry and reimbursement for the PSMA PET/CT scans.

Restaging with [18F]DCFPyL PET/CT (PSMA PET)
[18F]DCFPyL has a shelf- life of 12 hours and each centre will co-ordinate their process for delivery with the manufacturer, CPDC. [18F]DCFPyL will be administered intravenously at a dose of 325 MBq. The [18F]DCFPyL effective radiation dose equivalent for patients is estimated to be 0.0165 mSv/MBq. This translates to a dose of 5.4 mSv for the injected activity in this study. The CT component is for anatomic correlation and attenuation correction (non-diagnostic) and the radiation dose is 1.5-2.0 mSv. The total radiation dose is estimated to be 6.9-7.4 mSv. For the PET imaging, 60-120 minutes after injection of the [18F]DCFPyL the patient will be asked to void, and then will enter the PSMA PET/CT scanner for acquisition of the PET images with corresponding axial CT images obtained (for anatomic correlation and attenuation correction). Total scan time is approximately 60 minutes.

Following acquisition of the PSMA PET/CT images, interpretation by a local nuclear medicine physician will be conducted. Extent of disease within the prostate and at distant sites will be characterized and delineated on a standardized report. For sites of metastatic disease, suspicious lesions will be identified on PET based uptake characteristics (SUVmax) and correlated with the cross sectional CT imaging and assigned a 5-point suspicion of malignancy scale for each modality separately (+5 definitely malignant, +4 probably malignant, +3 equivocal, +2 probably not malignant, + 1 definitely not malignant). Central readers will be available to provide secondary reads in the case of cases where there is diagnostic uncertainty by the local reader. A report of the PSMA PET/CT will be supplied to the attending physician and a repeat management questionnaire will be completed that identifies any changes in the preferred management strategy based on the results of the [18F]DCFPyL imaging.

Confirmatory Biopsy
While not mandated by the study, men with restaging studies that suggest disease recurrence are encouraged to undergo biopsy confirmation if local salvage therapies are being considered. For example, those men for whom restaging suggests isolated intra-prostatic recurrence, confirmatory biopsy (either standard TRUS guided biopsy or MRI Guided biopsy) is typically recommended prior to undertaking local salvage therapy. Likewise, those patients with extra-prostatic disease identified on standard or experimental imaging who are being considered for salvage ablative therapies like SBRT for oligometastatic disease are encouraged to undergo confirmatory biopsy prior to salvage.

Follow-up and Outcomes
Individual sites are required to submit a formalized report from the staging PSMA PET scan to Cancer Care Ontario within 4 weeks of completion of the scan in order to obtain reimbursement for the scan. The report will identify the number and sites of recurrence identified on PSMA PET/CT imaging. Requesting physicians (Urologists and Oncologists) will be asked to complete a PSMA PET/CT management impact questionnaire to determine any change in the preferred management strategies (i.e. change from lesion directed therapy to hormone therapy) based on the information provided by [18F]DCFPyL imaging. Actual management delivered to individual patients after restaging including observation only, biopsy, local or distant salvage or systemic salvage will be assessed at 6 months post imaging through linkage to provincial health registries to track interventions and systemic therapy use.

OUTCOMES ASSESSMENT

Primary Objective PREP
To determine the proportion of men with sites of disease identified at the time of early biochemical failure in high risk disease in men treated with prostatectomy with or without adjuvant or salvage pelvic radiotherapy or hormone therapy as well as men treated with primary radiotherapy. This will be calculated as the number of men with at least 1 PSMA PET detected lesion that is scored as +5 (definitely malignant) or +4 (probably malignant) according to the local imaging read.

Primary Objective PREP Phase 2
To determine the proportion of men with sites of disease identified at the time of early biochemical failure in high risk disease in men treated with prostatectomy with or without adjuvant or salvage pelvic radiotherapy or hormone therapy as well as men treated with primary radiotherapy when PSMA PET/CT is used without routine prescreening with conventional imaging. This will be calculated as the number of men with at least 1 PSMA PET detected lesion that is scored as +5 (definitely malignant) or +4 (probably malignant) according to the local imaging read.

Secondary Objectives
1. The likelihood of disease detected on PSMA PET will be correlated with absolute PSA level at the time of PSMA PET as supplied on the eligibility form.
2. The proportion of men with oligometastatic recurrence (four or fewer sites including the prostate bed if positive) detected on PSMA PET will be calculated as the number of men with 4 or fewer lesions identified as +5 (definitely malignant) or +4 (probably malignant) divided by the number of men with any number of +5 or +4 lesions detected.
3. The number of men who have a change in management will be determined by comparison of the management questionnaire obtained at the time of registration to the repeat questionnaire performed after the PSMA PET scans are reported.
4. Actual management within 6 months will be determined through linkage to existing health information registries and will include:
    a. Delivery of radiotherapy (anatomic site, dose and fractionation) – Cancer Care Ontario
    b. Biopsy of suspected recurrences (anatomic site, histology) – Provincial pathology database
    c. Use of salvage lymph node dissections – CIHI
    d. Use of salvage hormonal therapy/androgen deprivation
5. PSA response will be examined by comparing 6 month PSA against PSA at the time of PSMA PET through the Ontario Laboratory Information Services and correlated with actual management as determined in 4.
6. Comparison of the detection rates of PSMA PET/CT when conventional imaging is used as part of eligibility criteria (PREP) versus when conventional imaging is omitted (PREP Phase 2)

ADVERSE EVENTS
This study will be conducted in accordance with Health Canada regulatory requirements and ICH Good Clinical Practice guidelines. Only adverse events (AEs) and Serious Adverse Events (SAEs) data attributable to the investigational imaging [18F]DCFPyL PET/CT will be tracked and reported.

Adverse Event Definitions
An Adverse Event (AE) is defined as any untoward medical occurrence in a patient who is administered a drug or biologic (medicinal product) or using a medical device; the event does not necessarily have a causal relationship with this treatment or usage. An AE can therefore be any unfavourable and unintended sign (including an abnormal laboratory finding), symptom, or disease temporally associated with the use of a medicinal (investigational) product, whether or not related to the medicinal (investigational) product. Each AE is to be classified by the Investigator as serious or non-serious.

A Serious Adverse Event (SAE) is any untoward medical occurrence that at any dose:
1. results in death
2. is life-threatening (i.e., immediate risk of death),
3. requires inpatient hospitalization or prolongation of existing hospitalization, except pre-planned hospitalizations required as part of a standard procedure or cancer treatment,
4. results in persistent or significant disability / incapacity,
5. is a congenital anomaly / birth defect

OR

6. is an important medical event, which is not immediately life threatening or requiring hospitalization, but may otherwise jeopardize the patient or may require intervention to prevent other outcomes specified in the above definition of SAE

An AE is unexpected when the nature or severity of the AE is not consistent with the applicable product information (i.e., Investigators Brochure for an unapproved investigational product or package insert/summary of product characteristics for an approved product). An AE is considered to be associated with the use of the drug if the attribution is classified as “Possible”, “Probable” or “Very Likely”.

Attribution Definitions

1. Unrelated: An AE which is not related to the use of the study drug.
2. Unlikely: An AE for which an alternative explanation is more likely (e.g. concomitant medication, concomitant disease), and/or the relation with time suggests that a causal relationship is unlikely.
3. Possible: An AE which might be due to the use of the study drug. An alternative explanation (e.g., concomitant medication, concomitant disease) is inconclusive. The relationship in time is reasonable; therefore the causal relationship cannot be excluded.
4. Probable: An AE which might be due to the use of the study drug. The relationship in time is suggestive (e.g., confirmed by a de-challenge). An alternative explanation is less likely (e.g., concomitant medication, concomitant disease).
5. Definitely Related: An AE, which is listed as a possible adverse reaction and cannot be reasonably explained by an alternative explanation (e.g., concomitant medications, concomitant diseases). The relationship in time is very suggestive (e.g. it is confirmed by a de-challenge and a re-challenge).

Adverse Event Reporting Criteria

AEs and SAEs will be considered related to study if they are related specifically to the [18F]DCFPyL PET/CT imaging. Progression of cancer and complications related to conventional imaging or cancer treatment (e.g. surgery, chemotherapy and/or radiotherapy) are expected and bear no relationship to the imaging protocol, therefore they will not be documented as AEs or SAEs for the purpose of this Registry. Deaths due to recurrent cancer are also expected and will not be reported as SAEs for the purpose of this Registry. Whenever possible, symptoms should be grouped as a single syndrome or diagnosis. The Investigator should specify the date of onset, grade, action taken with respect to the imaging protocol, corrective treatment or therapy given, outcome and his/her opinion as to whether there is a reasonable possibility that the AE was related to the imaging protocol. The severity of all AEs will be graded according to the NCI CTCAE, version 4. For each event, the highest severity grade attained since the last assessment period will be reported. If a CTCAE score does not exist, the Investigator should assess the event as Grade 1 (mild), Grade 2 (moderate), Grade 3 (severe), Grade 4 (life-threatening or disabling) or Grade 5 (causing death) to describe the maximum intensity of the AE.

Adverse Event Reporting Period

The AE reporting period for this study begins at the time of imaging and continues until 30 days after the imaging is completed. In the case of an SAE, the patient will be followed until clinical recovery or until progression has been stabilized or judged to be chronic.

Serious Adverse Event Reporting

Adverse events considered to be SERIOUS and UNEXPECTED and RELATED (that is, attributed as Possibly, Probably, or Very Likely) to the [18F]DCFPyL PET/CT imaging , must be recorded on a Serious Adverse Event Form and be reported by the site Investigator to the Sponsor, the REB, and regulatory authorities within the required timelines.

- Sponsor: within 24 hours of becoming aware of the event.
- OCREB: within 48 hours from the time the Investigator becomes aware of the event.
- Health Canada: within 7 days if SAE is fatal or life threatening, and 15 days if NOT fatal or life threatening.

All other SAEs should be reported within 5 days from the time when the site personnel became aware of the event.

Follow-up reports must be submitted to the Sponsor, the REB, and regulatory authorities when new information becomes available and not later than 5 days after the clinical site personnel became aware of the event. If an ongoing SAE changes in its intensity (Grade) or relationship to the Investigational Product, a follow-up SAE report should be sent immediately.

In the rare event that the Investigator does not become aware of the occurrence of an SAE immediately, the Investigator is to report the event to the REB, the Sponsor and regulatory authorities within 24 hours of notification of the event.

STUDY ADMINISTRATION

Ethical and Regulatory Standards

Informed Consent

It is the responsibility of the local Investigator or a person designated by the local Investigator and under the Investigator's responsibility, to provide each potential study patient, prior to inclusion in the study, full and adequate verbal and written information regarding the objectives and procedures of the study and the possible risks involved. The patient must be informed about their right to withdraw from the study at any time. The patient must be allowed adequate time to make an informed decision. Prior to a patient's participation in the study, the locally approved Information and Consent Form (ICF) must be signed, name filled in and personally dated by the patient or by the patient's legally acceptable representative, and by the person who conducted the informed consent discussion. A copy of the signed and dated written consent form document and any other written information will be provided to the patient. Written informed consent will be obtained from all potentially eligible patients prior to commencing any study procedures. Until the patient has been completely informed of the clinical trial, has freely consented to take part in the study and has signed and dated an informed consent form that has received documented approval by a licensed Research Ethics Board, no study related procedures can be performed.

Research Ethics Board (REB)

This Registry will utilize the Ontario Cancer Research Ethics Board (OCREB) as the Board of Record. Prior to the commencement of the Registry, the Investigator must submit this clinical trial protocol, the ICF document, Investigator Brochure or product labeling information, recruitment materials/process, patient questionnaires, and any other written information to be provided to study patients to the appropriate Ethics Committee (OCREB) and is required to forward to the sponsor a copy of the written and dated approval/favourable opinion signed by the Chairman with Ethics Committee (OCREB) composition. The clinical trial (study number, clinical trial protocol title, version number and version date), the documents reviewed (clinical trial protocol, Informed Consent Form, Investigator's Brochure, etc.) and the date of review should be clearly stated on the written (OCREB) approval/favourable opinion. Any amendments or modification to the study protocol and/or Informed Consent Form document must be submitted to and approved by the Ethics Committee (OCREB). The Ethics Committee (OCREB) should also be informed of any event likely to affect the safety of patients or the continued conduct of the study. Annual re-approval is required for as long as the study is open to patient accrual and/or patients are being followed and evaluated. The Ethics Committee (OCREB) must be informed when the study is closed or has been suspended.

Confidentiality of Registry Participant Records

The names and personal information of study participants will be held in strict confidence. Personal identifying information (name, date of birth, postal code, telephone number and OHIP number) will be sent to the local PET Centres participating in the registry to review participant eligibility and to CCO to track registry participants. This information, together with other information collected for the registry, will be kept confidential and securely transferred to Cancer Care Ontario and linked with routinely-collected information about the study participant's health care found in existing health-related databases (e.g., the Provincial Pathology Database, Canadian Institute for Health Information (CIHI), and the Ontario Laboratory Information Services).

The primary investigator at each participating site will maintain a confidential subject identification list (Master List) during the course of the study. Access to confidential information (i.e., source documents and patient records) is only permitted for direct subject management and for those involved in monitoring the conduct of the study (i.e., Sponsors, representatives of the REB, and regulatory agencies). The participant's name will not be used in any public report of the study.

All imaging data ([18F]DCFPyL PET/CT scans) and reports will be stored in the participating sites' hospital medical records and will be accessible to physicians within the participant's circle of care.

STUDY ORGANIZATION

Institutional Participation and Qualified Investigator (QI) responsibilities

The protocol will be conducted at six Ontario sites: London Regional Cancer Program, Princess Margaret Cancer Centre and Toronto Sunnybrook Cancer Centre, St. Joseph's Healthcare Hamilton, Ottawa Regional Cancer Centre and Thunder Bay Regional Cancer Centre. One Qualified Investigator (QI) will oversee the registry study at each clinical centre. Co-investigators at each Centre should include at a minimum a dually qualified nuclear medicine/radiology physician (alternatively separate nuclear medicine and radiology physicians) and urologist. The QI undertakes to enroll patients on the registry study in accordance with this protocol, ICH guidelines for Good Clinical Practice and the applicable national regulations and local REB requirements. The QI works with the co-investigators and may appoint other individuals, as he/she deems appropriate to assist in the conduct of the study. All appointed designates will be listed and provided to the sponsor. The appointed designates will be supervised by and under the responsibility of the QI. For the purpose of ensuring compliance with the clinical trial protocol, ICH GCP and applicable regulatory requirements, the QI agrees to permit study monitoring/auditing by or on the behalf of the sponsor, the institution and inspection by applicable regulatory authorities. The Investigator agrees to allow the auditors/inspectors to have direct access to his/her study records for review.

Registry Coordination

The Sponsor is responsible for the overall study management including finance and contracts, implementation of the study protocol logistics, patient allocation, data management, quality assurance. The Sponsor is responsible for monitoring study execution, particularly with regard to methodological aspects and ensuring each clinical centre adheres to the study protocol. There will be a data query process, visit management tracking and SAE tracking to ensure data is complete, accurate, of high quality and is reported/submitted according to required timelines. An in-house Ethics & Regulatory Affairs Officer will complete and submit the initial application to OCREB and facilitate the other centres' applications, ensuring required start-up documentation is obtained from each clinical centre prior to centre activation.

Data Safety Monitoring Committee

The investigational imaging agent, [18F]DCFPyL has undergone human testing with no adverse toxicity noted. As such a formal Data Safety Monitoring Committee will not be arranged for this study. In the case of any AEs, SAEs of grade 3 or higher attributable to the investigational imaging, accrual to the study will be suspended and the case in question reviewed by the Principal Investigators to identify any contributing or remedial causes before continuing accrual.

<u>STATISTICAL CONSIDERATIONS</u>

Statistical Analyses

Descriptive statistics will be used to summarize patient, tumour and treatment characteristics, along with endpoints. The primary endpoint will be described using proportions, along with a 95% exact confidence interval, and tested using an exact test of proportions. Secondary endpoints will be summarized using descriptive statistics, along with confidence intervals as appropriate. All tests and confidence intervals will be two-sided and statistical significance will be defined at the $\alpha=0.05$ level. No statistical adjustments will be performed for multiple testing; however, appropriate caution will be taken when inferring results from this study due to the fact that multiple tests will be

performed. Given the sample size, all tests and confidence intervals will be based on exact or non-parametric methods.

Null hypothesis
PSMA PET will detect sites of recurrence at the time of early biochemical failure after prior prostatectomy at a rate equal to that of conventional imaging.

Alternative hypothesis
PSMA PET will detect sites of recurrence at the time of early biochemical failure with a higher frequency as compared to conventional imaging.

Sample size calculation
Conventional imaging can be expected to detect recurrent disease in under 10% of men at the time of biochemical failure. Detection of sites of disease in 30% or more men would be considered a clinically meaningful improvement in performance compared to conventional imaging. For H0=0.10 and HA=0.30, a=0.05 and b=0.10, with a two-sided, one-sample test of proportion a minimum sample size of 56 men will be required in each cohort for initial registry analysis. Assuming approximately a 15% dropout a total of at least 65 men per cohort will be necessary or minimum 455 men overall for initial registry analysis. Those cohorts demonstrating positive results will remain open for further access while further regulatory and funding approval is sought.

Sample Size Revision, Cohorts 2, 3, 6
A recent meta-analysis by Perera et al (27) of 37 studies and 4790 patients focused on the performance of 68Ga-PSMA in primary staging as well as restaging of men with biochemical failure after radical prostatectomy or radiotherapy. They found a detection rate of 0.48 (PSA 0.2-0.49) to 0.97 (PSA>2) depending on the PSA at time of imaging following biochemical failure. In order to demonstrate that 18F-DCFPyL provides a similar detection rate as described by Perera et al for 68Ga-PSMA based agents, we will increase our sample size for cohorts 2, 3 and 6. To allow us to estimate the detection rate with a precision of +/-5% (with 95% confidence), assuming a detection rate of 60%, we will require 369 patients per cohort, 425 assuming a 15% dropout rate. With cohorts 1, 4, 5 and 7 remaining at 65 men each according to the original endpoint, this will result in a total sample size of 1535 men.

Sample Size Revision - PREP Phase 2
As per the Study Design for PREP Phase 2, the same Cohort sizes will be utilized in PREP Phase 2. Specifically, Cohorts 2,3,6 will have target accruals of 425 each for Phase 2. Cohorts 1,4,5,7 will remain at 65 each for Phase 2. As per PREP, all cohorts will remain open to accrual as long as any individual level has not reached the accrual target.

REFERENCES

1. Heidenreich A, Bastian PJ, Bellmunt J, Bolla M, Joniau S, van der Kwast T, et al. EAU guidelines on prostate cancer. Part II: Treatment of advanced, relapsing, and castration-resistant prostate cancer. European urology. 2014;65(2):467-79.
2. Krahn M, Bremner KE, Tomlinson G, Luo J, Ritvo P, Naglie G, et al. Androgen deprivation therapy in prostate cancer: are rising concerns leading to falling use? BJU international. 2011;108(10):1588-96.
3. Crook JM, O'Callaghan CJ, Duncan G, Dearnaley DP, Higano CS, Horwitz EM, et al. Intermittent androgen suppression for rising PSA level after radiotherapy. N Engl J Med. 2012;367(10):895-903.
4. Weichselbaum RR, Hellman S. Oligometastases revisited. Nat Rev Clin Oncol. 2011;8(6):378-82.
5. Crehange G, Roach M, 3rd, Martin E, Cormier L, Peiffert D, Cochet A, et al. Salvage reirradiation for locoregional failure after radiation therapy for prostate cancer: who, when, where and how? Cancer Radiother. 2014;18(5-6):524-34.
6. Moreno A, Albiach CF, Soria R, Vidal VG, Gomez RG, Antequera MA. Oligometastases in prostate cancer: restaging stage IV cancers and new radiotherapy options. Radiation Oncology. 2014;9.
7. Ost P, Bossi A, Decaestecker K, De Meerleer G, Giannarini G, Karnes RJ, et al. Metastasis-directed Therapy of Regional and Distant Recurrences After Curative Treatment of Prostate Cancer: A Systematic Review of the Literature. European urology. 2014.

8. Reeves F, Murphy D, Evans C, Bowden P, Costello A. Targeted local therapy in oligometastatic prostate cancer: a promising potential opportunity after failed primary treatment. BJU Int. 2014.
9. Palma DA, Salama JK, Lo SS, Senan S, Treasure T, Govindan R, et al. The oligometastatic state-separating truth from wishful thinking. Nature reviews Clinical oncology. 2014.
10. Crawford ED, Stone NN, Yu EY, Koo PJ, Freedland SJ, Slovin SF, et al. Challenges and recommendations for early identification of metastatic disease in prostate cancer. Urology. 2014;83(3):664-9.
11. Afshar-Oromieh A, Haberkorn U, Schlemmer HP, Fenchel M, Eder M, Eisenhut M, et al. Comparison of PET/CT and PET/MRI hybrid systems using a 68Ga-labelled PSMA ligand for the diagnosis of recurrent prostate cancer: initial experience. European journal of nuclear medicine and molecular imaging. 2014;41(5):887-97.
12. Bauman G, Belhocine T, Kovacs M, Ward A, Beheshti M, Rachinsky I. 18F-fluorocholine for prostate cancer imaging: a systematic review of the literature. Prostate Cancer Prostatic Dis. 2012;15(1):45-55.
13. Cho SY, Gage KL, Mease RC, Senthamizhchelvan S, Holt DP, Jeffrey-Kwanisai A, et al. Biodistribution, tumor detection, and radiation dosimetry of 18F-DCFBC, a low-molecular-weight inhibitor of prostate-specific membrane antigen, in patients with metastatic prostate cancer. Journal of nuclear medicine : official publication, Society of Nuclear Medicine. 2012;53(12):1883-91.
14. Afshar-Oromieh A, Zechmann CM, Malcher A, Eder M, Eisenhut M, Linhart HG, et al. Comparison of PET imaging with a (68)Ga-labelled PSMA ligand and (18)F-choline-based PET/CT for the diagnosis of recurrent prostate cancer. European journal of nuclear medicine and molecular imaging. 2014;41(1):11-20.
15. Morigi JJ, Stricker PD, van Leeuwen PJ, Tang R, Ho B, Nguyen Q, et al. Prospective Comparison of 18F-Fluoromethylcholine Versus 68Ga-PSMA PET/CT in Prostate Cancer Patients Who Have Rising PSA After Curative Treatment and Are Being Considered for Targeted Therapy. J Nucl Med. 2015;56(8):1185-90.
16. Albisinni S, Artigas C, Aoun F, Biaou I, Grosman J, Gil T, et al. Clinical impact of 68 Ga-prostate-specific membrane antigen (PSMA) positron emission tomography/computed tomography (PET/CT) in patients with prostate cancer with rising prostate-specific antigen after treatment with curative intent: preliminary analysis of a multidisciplinary approach. BJU Int. 2016.
17. Dietlein M, Kobe C, Kuhnert G, Stockter S, Fischer T, Schomacker K, et al. Comparison of [(18)F]DCFPyL and [(68)Ga]Ga-PSMA-HBED-CC for PSMA-PET Imaging in Patients with Relapsed Prostate Cancer. Mol Imaging Biol. 2015;17(4):575-84.
18. Evans WK, Laupacis A, Gulenchyn KY, Levin L, Levine M. Evidence-based approach to the introduction of positron emission tomography in ontario, Canada. J Clin Oncol. 2009;27(33):5607-13.
19. Valliant J. Centre for Probe Development and Commercialization 2015 [Available from: http://www.imagingprobes.ca/.
20. Chin JL, Touma N. Current status of salvage cryoablation for prostate cancer following radiation failure. Technol Cancer Res Treat. 2005;4(2):211-6.
21. Palma DA, Haasbeek CJ, Rodrigues GB, Dahele M, Lock M, Yaremko B, et al. Stereotactic ablative radiotherapy for comprehensive treatment of oligometastatic tumors (SABR-COMET): study protocol for a randomized phase II trial. BMC cancer. 2012;12:305.
22. Menard C, Smith IC, Somorjai RL, Leboldus L, Patel R, Littman C, et al. Magnetic resonance spectroscopy of the malignant prostate gland after radiotherapy: a histopathologic study of diagnostic validity. Int J Radiat Oncol Biol Phys. 2001;50(2):317-23.
23. Menard C, Susil RC, Choyke P, Gustafson GS, Kammerer W, Ning H, et al. MRI-guided HDR prostate brachytherapy in standard 1.5T scanner. Int J Rad Oncol Biol Phys. 2004;59(5):1414-23.
24. Chen Y, Pullambhatla M, Foss CA, Byun Y, Nimmagadda S, Senthamizhchelvan S, et al. 2-(3-{1-Carboxy-5-[(6-[18F]fluoro-pyridine-3-carbonyl)-amino]-pentyl}-ureido)-pen tanedioic acid, [18F]DCFPyL, a PSMA-based PET imaging agent for prostate cancer. Clin Cancer Res. 2011;17(24):7645-53.
25. Broughman JR, Chen RC. Management of Node-Positive and Oligometastatic Prostate Cancer. Semin Radiat Oncol. 2017;27(1):79-86.
26. Parker WP, Evans JD, Stish BJ, Park SS, Olivier K, Choo R, et al. Patterns of Recurrence After Postprostatectomy Fossa Radiation Therapy Identified by C-11 Choline Positron Emission Tomography/Computed Tomography. Int J Radiat Oncol Biol Phys. 2017;97(3):526-35.
27. Kane CJ, Amling CL, Johnstone PAS, Pak N, Lance RS, Thrasher JB, et al. Limited value of bone scintigraphy and computed tomography in assessing biochemical failure after radical prostatectomy. Urology. 2003;61(3):607-11.

28. De Visschere PJL, Standaert C, Futterer JJ, Villeirs GM, Panebianco V, Walz J, et al. A Systematic Review on the Role of Imaging in Early Recurrent Prostate Cancer. Eur Urol Oncol. 2019;2(1):47-76.
